# Intimate partner violence during lockdown in Tuscany, Italy: Economic or confinement-related shocks?

**DOI:** 10.1371/journal.pone.0349889

**Published:** 2026-06-24

**Authors:** Francesca Bettio, Fernando Flores Tavares, Elisa Ticci

**Affiliations:** 1 University of Siena, Department of Economics and Statistics, Siena, Italy; 2 University of Siena, Department of Political and International Sciences, Siena, Italy; Caleb University, NIGERIA

## Abstract

We revisit the issue of Intimate Partner Violence (IPV) during the COVID-19 pandemic asking three questions: whether IPV worsened with lockdown, what pandemic-specific ‘shocks’ had the greatest impact, and how the results change when different measures of IPV are used. The large telephone survey we leveraged for this purpose was conducted in 2021 in the Italian region of Tuscany as part of a mixed-method research project on IPV during the first lockdowns, and it is, to our knowledge, the only (locally) representative survey on IPV during lockdowns conducted in Italy. Subjective evidence from the survey shows that, on balance, IPV worsened in frequency or severity or both. Econometric evidence suggests that parental overburden due to the presence of minors had the largest influence, followed by job loss, whereas we were not able to discern a significant influence for confinement in crowded space lacking privacy. Finally, and unsurprisingly, we found that using a fuzzy measure of violence outcomes that accounts for severity and intensity as well as prevalence of violence allows us to discern between shocks like job loss that primarily influenced the occurrence of violence without significantly influencing its harshness. Our empirical strategy principally relies on the exogeneity of pandemic-specific shocks to attribute causal interpretation to our estimates. However, our dependent variables (IPV outcomes) are binary or fractional, and endogeneity of some control covariates cannot be ruled out. To address these issues, we estimate average marginal effects using a two-step Control function (CF) approach combined with a quasi-likelihood method.

## 1. Introduction

Since the COVID-19 pandemic, the literature on violence against women - Intimate Partner Violence (IPV) in particular – has reached massive proportions. A large part of the contributions are descriptive and/or based on quickly gathered online data [1–4] or administrative sources like helpline calls or police records [5,6] yielding a rich and timely but somewhat fragmented picture.

In this paper, we revisit the issue of IPV during the pandemic leveraging an ad-hoc survey conducted in 2021 in the Italian region of Tuscany as part of a mixed-method research project on IPV (henceforth ‘our survey’ or ‘PANGEA survey’). The survey focuses on lockdown periods between March 2020 and April 2021 in Italy, one of the countries first and worst hit by the pandemic, where strict restriction measures were implemented [7]. Though local, the survey is representative of the Tuscan population, which was 3.66 million in December 2021, not much lower than the population of Croatia and almost twice that of Latvia.

We contribute to the literature along three main lines. First, we focus our investigation on the role of potential triggers that have been considered across disciplines under different theoretical approaches and for which a case of exogeneity can reasonably be made on account of the pandemic. These are, respectively, loss of job due to COVID, parental overburden due to the presence of minors during lockdowns, and being confined to crowded living space with no privacy during lockdowns. Economists call ‘shocks’ events like loss of job and income due to an unanticipated occurrence, and we argue that even normal life events like living with children or in crowded space can be treated as shocks when unanticipated events shape their outcomes. Factors contributing to IPV are often viewed as ‘stressors’ by psychologists, especially in the context of natural disasters, and variants of Hill’s family stress theory [8] have become popular also among sociologists. [9,10], for example, recently proposed this theory as a unifying framework to study domestic violence during the pandemic. In what follows we refer to job loss, parental overburden and confinement in overcrowded space as ‘shocks’ in order to emphasize their nature of unexpected events, hence of exogenous source of potential stress.

Job loss for both women and men has been linked to higher rates of IPV in normal times [11], but loss of job due to COVID can be treated as an exogenous economic shock if only because its sectoral distribution followed pandemic-specific regulations (for an in-depth analysis of the effects of the pandemic on Italian workers, refer to [12,13]). The presence of children has long been considered a correlate of IPV, albeit with mixed findings, but the parental overburden caused by the combination of home schooling and a practically 24-hour stay-at-home schedule was a novelty brought by COVID which deserves specific investigation as a potential source of conflict and aggression (see also [14] on school closures as a “disruptive exogenous shock” to family life). Finally, confinement meant living in crowded space in certain households, with no privacy. Crowding can increase family tensions, conflict, and aggression at any time, but the pandemic made it difficult or impossible to escape and hindered the capacity of victims to organize support. Therefore, while using the occurrence of COVID-19 as a natural experiment validating causal inference is not always considered appropriate [15], the pandemic may safely be exploited to strengthen the assumption of exogeneity for COVID-induced, potential IPV shocks, though it should be stressed that whatever impact is estimated need not be generalizable to ‘normal times’.

Our second contribution is measurement of outcomes. Any attempt to identify the links between IPV and its potential shocks hinges on accurate measurement of IPV outcomes. We consider two different measures. The first is prevalence, namely the percentage of women in the population who have experienced at least one episode of any abuse in a given period. Prevalence is traditionally computed for different types of violence (psychological, physical, sexual), and by virtue of its conceptual and computational simplicity, it has become standard in the literature. Because prevalence ignores the intensity and severity of violence, the need for a comprehensive measure is often voiced, including by international organizations [16–18]. A fuzzy IPV index was recently developed by [19] to account for intensity and severity in addition to prevalence, and we use this index as our second IPV measure. We show that the results and policy implications of the two measures differ in non-trivial ways.

As a final contribution, we bring additional evidence in support of the claim that, on balance, the pandemic was associated with a worsening of IPV in terms of frequency and/or severity.

The corresponding questions we address are as follows. Firstly, we reconsider the frequent claim in the literature that IPV increased during the pandemic. Although our data do not allow for a direct comparison of outcomes before and during the pandemic, we document that a significant number of women abused during lockdowns rated their IPV experience as being ‘worse’ (unprecedented or more frequent or more severe or all of these). Our second question concerns the effect on IPV of the three COVID-induced shocks we identified during lockdowns. As noted, the shocks are job loss for at least one partner, parental overburden due to living with children under 18 during lockdowns, and living in crowded space with no privacy during lockdowns. Since our dependent variables (IPV outcomes) are binary or fractional, our strategy to address endogeneity issues arising from covariates is to estimate average marginal effects using a two-step control function (CF) approach combined with a quasi-likelihood method.

Our final question is how IPV outcomes change if a comprehensive measure of violence against women is used instead of simple prevalence which, as noted, does not distinguish between women having suffered just one ‘light’ abuse and those having suffered frequent or multiple and/or severe abuses. While the general purpose in this case is to gain awareness of partial or distortive indications yielded by incomplete measurement, the specific purpose is to revisit existing knowledge about the differential impact of the factors involved in IPV during natural disasters.

The paper is structured as follows: the next section selectively reviews the vast literature already in existence, followed by essential information in Section 3 to contextualise this research. A detailed description of the data source is given next (Section 4) together with an overview of IPV incidence and the victim’s profile. The empirical strategy is illustrated at length in Section 5, and the findings are discussed in Section 6. A concluding summary wraps up the findings in Section 7 while also discussing implications and limitations.

## 2. Literature review

Expectations of an adverse impact of the pandemic on IPV follow from the historical experience of other natural disasters [20–24]. Limiting the list of citations to a few surveys and meta-studies [25–28], all of these concur that, on the whole, IPV and domestic violence increased during the pandemic.

In general, heightened risks of violence are associated with the stress factors that accompany disastrous events [29–33]. As noted by [34], however, earlier empirical evidence on whether stress from disasters actually causes IPV was not conclusive, though disasters have traditionally been associated with an escalation in the severity of violence and violent attitudes. Stress can originate from economic shocks brought on by the pandemic. With some exceptions that we now highlight, most existing studies on economic shocks exploited selected administrative data sources or online surveys conducted during the pandemic and not necessarily representative. Confining attention to advanced countries, the internet-based survey conducted by [35] noted that physical IPV perpetration and victimization were more common among those who lost their job. The online survey of [4] found that financial difficulties since the beginning of the COVID-19 pandemic were correlated with increasing first-time incidents of IPV or increased severity of IPV for those with prior experience of abuse. The large online survey of Australian women by [3] indicated that the likelihood of experiencing physical and sexual abuse was higher for women with no prior experience of violence who reported increased financial stress. [1] surveyed Canadian women online and showed that worries about the impact of COVID-19 on ability to meet financial obligations and essential needs were associated with greater concern about the impact of COVID-19 on family stress and domestic violence. Based on an online survey conducted in the US in the early months of COVID-19, [36] found that loss of household income during the pandemic was associated with near doubling of the frequency of family violence, mainly verbal abuse.

Only a few studies are based on representative surveys and/or exploit the exogeneity of COVID-related economic shocks to assign causal validity to the effects on IPV/domestic violence, but their findings are not entirely consistent. According to [37], in Spain not only job loss but also concerns about losing paid work or being on temporary layoff following the pandemic contributed to the surge in IPV. The authors found that IPV rose for all types of violence (physical, sexual and psychological) and the impact was significant when both members of the couple were economically affected or, in line with the male backlash hypothesis, when the male but not the female partner was affected. In Germany, a survey of 3,818 partnered women analysed by [38] found a higher likelihood of IPV for women in couples in which one or both partners experienced temporary or complete job loss due to the pandemic. [39] focused on job loss, and although their study concerns Peru, not a high-income country, it is worth mentioning here because the findings underline the importance of actual economic distress caused by job loss and dispute the role of mere economic anxiety brought on by the pandemic. However, according to [5], a different picture emerges from labour market, crime and mobile device tracking data at city level in the United States, where they found that local unemployment reduced domestic violence between January 2019 and November 2020.

Turning now to studies focusing on family factors associated with IPV, psychologists, sociologists and criminologists have long investigated these factors building on Hill’s seminal contribution to family stress theory as well as on popular conceptualizations of parenting/parental stress [40,41]. Although the pre-pandemic literature has examined parental stress primarily in connection with violence against children, IPV too has been found to correlate positively with the presence of children in ‘normal times’ (e.g., because women with children find it more difficult to quit a violent relationship [42–45]). With the onset of the pandemic, evidence that partners confined at home with children were at increasing risk of parenting stress or mental health deterioration revived interest in child maltreatment [24,46] rather than IPV. Studies considering IPV viewed the presence of children as an exacerbator of economic factors [2,4,37] or showed an inconsistent association with IPV [47]. We view the presence of minors during confinement as a potentially important pandemic-specific source of stress, hence a shock on its own. Parents faced a phenomenal increase in childcare and workload, especially when home-schooling replaced formal schooling. In their revisitation of family stress theory to interpret COVID-specific aspects of domestic violence, [10] argue that parents are likely to have experienced unprecedented daily stress associated with the challenges of home-schooling, as also noted by [48] for the Great Recession. Difficulties in balancing paid work and family or less satisfaction with family life have in fact been documented for several countries, especially among employed women with young children [49 for Italy; 14 for Germany]. Qualitative evidence from in-depth interviews conducted to complement the PANGEA survey (see section 3) suggests that female partners’ requests to redefine the traditional division of care workload during confinement sometimes turned bargaining into conflict that led to violent reactions when the male partner held strong traditional gender views [50]. More generally, the pandemic offered new opportunities for threats and forms of control involving children: for example, fear that children could spread the virus was exploited to prevent mothers from taking them outside [50]. The findings by [38, p. 13–14] are in partial disagreement: the authors could not confirm that reduced childcare capacity was a driver of IPV during 2020 lockdowns in Germany, but they report that help requests for IPV were actually higher during the first lockdown in States with higher childcare capacity. Moreover, their analysis is confined to toddlers whereas we would expect older children to have been a source of parental overburden equally if not more than toddlers.

The onset of psychological stress and aggressive behaviour in response to crowding and the absence of privacy is a topos in psychology and health-related disciplines, for example in relation to the conditions facing astronauts, jail inmates and certain patients on mandatory confinement in narrowly spaced wards (see, for example, [51,52]). Unsurprisingly, therefore, several contributions on domestic violence during the pandemic view overcrowding and lack of privacy during confinement as a potential cause of IPV or an aggravating factor. However, we only found few studies focusing specifically on space- and privacy-related issues in relation to aggressive behaviour during COVID, and fewer still focusing on IPV. The study by [53] is worth mentioning as a partial exception because it specifically investigates the relationship between, on the one hand, aggression, and, on the other hand, residential density, subjective crowding and perceived lack of privacy during national lockdowns in the UK. The authors found a stronger association with aggression for subjective crowding than for residential density, as well as a statistically significant association between aggression and perceived lack of privacy. Also, [54] focused on a low-income, high-violence neighbourhood in the US (New Orleans) and found that areas with parks were associated with reduced pandemic related distress, whereas areas with a high density of alcohol outlets and crowded streets were associated with greater distress. All this suggests that certain characteristics of the living space in which partners and their families were confined may have played an independent role in the surge of IPV. Overall, however, this is a poorly investigated issue.

Other sources of COVID-specific shocks frequently found in the literature include social isolation and the virus itself. According to [35], for example, being tested and testing positive to COVID-19 was correlated with higher risk of experiencing IPV, especially psychological violence. However, since practically the entire population under confinement was exposed to these risks at least once, and since differential exposure is hard to measure retrospectively, it would have been especially problematic to isolate the impact of these factors with the kind of survey we conducted. We can only assume that social isolation and fear of testing positive were largely common to the women in our sample and their partners.

Finally, heterogeneity of IPV outcomes based on personal and social/group characteristics is a common theme across all strands of the literature. The US literature, in particular, documents the significance of belonging to marginalized, immigrant communities in the case of women [4,55,56]. This also emerges from the profile of abused women in our survey (Section 4).

## 3. The context

Tuscany is of special interest for the study of IPV during the pandemic. As a region of Central Italy, hence very close to the epicentre of the first wave, Tuscany is one of the areas in Europe where non-pharmacologic measures for COVID-19 mitigation were first introduced and proved exceptionally stringent. The first national lockdown period was enforced in March 2020 and lasted two months. Restrictions included nationwide closure of schools, workplaces (except essential services) and public places, stay-at-home orders, self-isolation if ill, and quarantine if tested positive [57]. In May 2020, the Government allowed activities to gradually resume, but starting in November, it implemented locally differentiated restrictions based on local indicators. Tuscany, in particular, reintroduced stricter measures and local lockdowns in November-December 2020 and March-April 2021.

The social and economic costs were high, though mitigated by extraordinary measures including income support schemes and unemployment benefits: in 2020 the GDP of Tuscany dropped by 12 pp., 3 pp. more than the drop in national GDP, and more than had occurred from 2008 to 2009 amidst the global financial crisis [58].

According to pre-pandemic survey estimates from the National Statistical Institute for 2014 Tuscany [59], 12 months prevalence of violence from the current partner for women 16–70 years old was sensibly lower than the national average: figures for the region ranged from 0.2% (physical violence) and 0.9% (sexual violence) up to 4.5% (economic violence) and 11.3% (belittling and verbal abuse). Anti-violence centres providing a variable mix of services to abused women – from support and counselling to shelter – have been systematically mapped and monitored after the pandemic, and the figures for Tuscany in 2022 were broadly in line with national figures: between 0.11 and 0.16 centres per 10000 women operated in the region, against an overall average of 0.13 for Italy [60]. Like other regions in Northern and Central Italy, however, Tuscany suffered disproportionately from the pandemic restrictions imposed to some of the activities of the centres: the decline in the number of women accessing these services during the initial lockdown was more pronounced in Northern and Central regions than in other parts of Italy [61].

In Italy, like in other countries, the impact of the pandemic on violence against women has been primarily investigated by analysing data from the police force and anti-violence centres, including shelters and helpline calls. As is well known, however, it may not be easy to disentangle the influence that information campaigns or restricted access to services during the pandemic exercised on IPV data from administrative sources. Like in other parts of the world, the number of calls to helplines peaked when lockdowns coincided with massive information campaigns, and reports to the police from victims perceiving imminent danger increased during mobility restrictions and declined during their relaxation.

In view of all this, a survey administered to a representative sample of the population after the event of interest took place (lockdowns) has the advantage of avoiding source-specific distortions due to the pandemic while also reducing the risk of lower disclosure due to the physical presence of the cohabiting partner during confinement.

## 4. The data

### 4.1. Data collection and sample

As noted, the PANGEA data was gathered for inter-disciplinary, mixed-method research. Quantitative data was collected through a large cross-sectional survey with retrospective questions targeting the female adult population domiciled in the region of Tuscany and living with their partners during lockdowns.

To complement the quantitative findings and enrich their interpretation, qualitative data was collected by re-interviewing (willing) interviewees having reported abuse during lockdowns as well as by extending the survey to abused women who had sought shelter in anti-violence centres in Tuscany and had not been part of the survey first round. The findings from qualitative analysis are discussed at length elsewhere [50] but are selectively recalled here where relevant.

The data collection took place in three rounds: from August 17^th^ to September 18^th^, 2021 (3000 interviews); from November 22nd to December 17^th^, 2021 (743 interviews); and from November 5^th^, 2022 to May 30^th^, 2023 (35 interviews in anti-violence centres). In the first round of data collection, the interviewers administered the questionnaire by Computer-Assisted Telephone Interview (CATI). In the second round, a combination of CATI and Computer-Assisted Mobile Interview (CAMI) was used to reduce the bias of under-representation in the sample of those not appearing in telephone directories and those who do not have landline phones (typically young women). The third round oversampled actual victims by interviewing 35 women who had suffered from intimate partner violence during lockdowns and had sought shelter in anti-violence centres in Tuscany. These interviews were based on a semi-structured questionnaire that added open-ended questions to the closed questions of the CATI questionnaire. The oversampling technique is recommended when the group of interest tends to elude standard sample surveys on the general population [62].

The PANGEA survey questionnaire included 45 closed-ended questions (see S1 Table for list). The entire project, including the questionnaire, was approved by the Comitato per l’Etica Clinica dell’Azienda Ospedaliero – Universitaria Senese (COMEC) of the University of Siena. Before the interview started, respondents were informed that their data was strictly confidential, and that privacy and anonymity were guaranteed. All interviews were conducted in Italian by selected and trained interviewers by Numeria Srl, the company that carried out the survey. Interviews began only after obtaining informed verbal consent from each participant. This consent was documented through authorised audio recordings made and stored by the interviewers. All the data was anonymised by Numeria, so the authors did not have access to any information that could identify the women interviewed during or after data collection. The terms of the PANGEA project granted access to the anonymised data to the authors and other members of the project with no expiration. Therefore, we accessed the data for this research multiple times from September 15^th^, 2021, until March 31st, 2026.

We developed the questions using the classification and language of the National Statistical Institute ‘Women’s Safety Survey’ [56,63], the European Fundamental Rights Agency Survey on women’s well-being and safety [64], and the EU survey on gender-based violence against women and other forms of inter-personal violence (EU-GBV) [16]. Accordingly, we classified violence against women into three types – psychological, physical, and sexual – with an added subdivision between COVID-related psychological violence and general psychological violence. Table S1 in S1 Appendix lists the individual acts of violence in each type. To address the missing data from variables with no response, we used stochastic imputation methods. Table S3 in S1 Appendix provides further details about the imputation.

The sampling strategy hypothesized a 10% IPV prevalence, a figure broadly in line with previous estimates (see previous section). The quantitative survey contacted 35709 women, 3778 of whom agreed to be interviewed. The sample from the first two rounds of 2021 (3743 observations) underwent filtering and qualitative analysis of the interviews which led to the validation of 3599 observations. After adding the 35 observations from the CAVs, the final available sample comprised 3634 women between 18 and 75 years of age. Although the response rate was modest, representativeness was ensured through careful stratified sampling and post-survey weighting. Specifically, sampling was stratified by province and implicitly by municipality size to ensure geographical representativeness. Subsequently, we weighted all 3599 observations from the first two rounds according to the official age distribution of women in Tuscany across five age groups (18–34, 35–44, 45–54, 55–64, 65–75) and the proportional female population across the ten Tuscan provinces. Finally, since the additional observations in the third round had an equal probability of experiencing violence as in the prior rounds, we redistributed the weights of those who experienced violence proportionally, to ensure that the relationship between violence and non-violence remained unchanged. Therefore, the sample is representative of the population in terms of age distribution among women and their geographical distribution across provinces.

For the purposes of this paper, we restricted the sample to working-age women (18–64) who are not pensioners, as our research focus is specifically on intimate partner violence during working age. This restriction resulted in a working sample comprising 2061 observations.

### 4.2. Measurement and overview

*IPV measures and findings.* As anticipated, a new feature of this study is the way IPV outcomes are measured. We used two measures, the conventional prevalence of violence whereby women are considered abused if they suffer a single act of violence, irrespective of intensity and severity, and a fuzzy index which also accounts for severity and intensity. This index was derived by [19] and exploits fuzzy logic under the following premises. First, intensity is gauged by the frequency with which each abuse occurs. Second, severity is understood as socially perceived severity and is proxied by the inverse probability of occurrence of a certain abusive act, based on the assumption that the effort societies put into fighting adverse outcomes is broadly commensurate with the perceived severity of these outcomes. This assumption is discussed at length by the proponents of the index who also validated the resulting severity scale by comparing it with existing scales, usually much more costly to compute and less fine grained. Third, acts of violence are ranked by severity only if they pertain to the same type of violence (e.g., psychological or physical or sexual), since ranking across types is problematic. Fourth, since certain acts of violence tend to occur together, the severity scale based on inverse prevalence is corrected for correlation between acts of violence in order to counter problems of measurement and avoid redundancy. Under these premises, fuzzy logic can be used to combine prevalence, intensity, and severity into type-specific indexes that are computed for each individual and can be aggregated across individuals. Further aggregation of type-specific indexes into an overall index can be controversial because of the difficulty of comparing severity across violence types. The most transparent option, and the one we adopt in this paper, is to take a simple average, which is equivalent to abstaining from ranking violence types in terms of severity, i.e., physical violence is not viewed as more severe, per se, than other types as it all depends on the specific abuse that is perpetrated.

[19] formalized the construction of the index, and applied it to the first Europe-wide survey of violence against women carried out by the Fundamental Rights Agency (FRA) [64]. A Stata code to actually implement the index is available on GitHub [65].

The fuzzy index we present here uses the severity scale obtained by [19]. Given sufficient cultural homogeneity between the population of Tuscany and that of the European Union as a whole, using a scale which is based on a very large sample has clear advantages. However, since differences between the FRA questionnaire and our own required some amendments, we report the revised scale in S1 Appendix. Moreover, the present index proxies intensity with multiplicity of abuses rather than frequency, i.e., with the number of different abuses of a given type the woman reported. This is because the PANGEA questionnaire traded off investigation of mere frequency with that of perceived, overall change in IPV (see Section 6).

Table 1 summarizes the evidence from our sample on the distribution of IPV by types in terms of prevalence and fuzzy index values. Average prevalence refers to the whole population while average fuzzy index values are also reported for the subgroup of abused women. Of the 2061 women in our working sample, approximately 150 experienced abuse at least once from their partner during lockdowns. After applying sampling weights (to correct for oversampling and to account for the age and geographical distribution of women, as noted), the overall prevalence is 5.7%, which corresponds to approximately 60,245 women in the population (considering the total population of women between 18 and 64 minus the recipients of pension: data available at Istat [66]). Psychological violence was the most widespread as it affected nearly all self-reported victims (5.4% prevalence). Physical violence affected less than half the victims (2.1% prevalence) while sexual violence was reported by 1.3% of women. Lockdown conditions made it easier for the partner to exercise old and new forms of control, like preventing the woman from working, contacting friends and family and even contacting doctors, clinics and hospitals, and more generally from leaving home (as far as allowed by confinement measures). Nearly 40% of the victims reported suffering at least one such form of control.

**Table 1:** Prevalence and Fuzzy index of IPV during lockdown, by type.

	Panel A: Victims, prevalence, and mean number of violent acts		Panel B: Fuzzy Index of Violence	
	Prevalence (%)	Distinct violent acts experienced by victims (Mean)	Censored fuzzy index (Mean value for victims of specific violence type)	Fuzzy index (Mean value for all women)
All violence types	5.73	5.45	0.182	0.010
	[0.47]	[0.50]	[0.182] (0.180)	[0.001] (0.013)
Psychological	5.43	3.90	0.255	0.014
	[0.46]	[0.28]	[0.224] (0.251)	[0.224] (0.017)
Physical	2.14	3.50	0.360	0.008
	[0.29]	[0.32]	[0.364] (0.351)	[0.001] (0.010)
Sexual	1.32 [0.25]	1.91 [0.14]	0.470 [0.370] (0.472)	0.006 [0.001] (0.008)
Observations	2,061			2,061

Notes: Standard errors in brackets. Outcomes with sampling weights and imputed values (except for the last row). For Panel B, non-weighted results in parenthesis.

The ranking among IPV types stays the same when the fuzzy index is computed for the entire female population of working age women while reversing when computed only for women having experienced abuse. In this latter case, the index is not influenced by prevalence and gauges severity and intensity of abuses: its value reaches 0.47 for sexual violence (out of 1) followed by physical (0.36) and psychological violence (0.26). The overall fuzzy index value for abused women (0.18) is lower than any type-specific value since not all victims suffered from all types of violence.

How do the above figures compare with those from other studies on IPV during lockdown? Concerning prevalence, there is unfortunately no exhaustive answer. According to the 2014 ISTAT estimates for Tuscany we reported earlier [59], 12 months prevalence before the pandemic was lower than our estimates during lockdowns for sexual and physical violence but higher for psychological violence. However, in view of the numerous differences between our questionnaire and that used by ISTAT in 2014 (e.g., age groups or inclusion of non-partners among potential perpetrators) a before – after COVID comparison is problematic, except perhaps for the indication that physical and sexual violence are likely to have increased during the pandemic.

As concerns international comparisons, only a minority of studies based on telephone surveys can be taken as reference, especially those for European countries. [67] conducted a telephone survey in January 2021 on a representative sample of 1541 Portuguese women to investigate episodes of violence since March 2020 and found that 4.9% of respondents experienced violence for the first time in that period. This is a lower figure than ours, but the difference is easily explained by the exclusion of women with a previous history of violence as well as by a much shorter questionnaire in the Portuguese survey. A higher figure (9.4%) than ours is instead reported by [68] based on a large, nationally representative telephone survey carried out in Germany between February and March 2021 to interrogate women about their experience in the previous 12 months. Overall, however, the above differences are still small with respect to those resulting from online surveys. For example, an online survey for Portugal [69] found a 13.7% prevalence of domestic violence, mostly psychological, between April and October 2020. Back to Germany, a large online survey found an overall prevalence of 8% but for a much shorter reference period (April to May 2020) [38]. One factor that may have amplified differences between online and telephone surveys is that recent experiences of violence tend to be associated with a higher reporting rate [70] and online surveys were often conducted during or soon after the worst lockdown episodes. Moreover, because online surveys are self-administered, they often provide a sense of anonymity that encourages more confident disclosure compared to telephone surveys where the presence of an interviewer can trigger social desirability bias [71]. Additionally, online surveys typically have lower rates of item nonresponse than telephone surveys [72].

On balance, we cannot rule out underestimation of psychological violence by our survey. While there is still insufficient knowledge about the influence that different methods of data collection may have on IPV recall bias [73], our qualitative evidence suggests that psychological abuse may have been comparatively underreported in the PANGEA survey. The qualitative investigation we conducted by re-interviewing women with experience of abuse during the lockdown revealed a tendency for some of them to minimize and even disavow ‘lighter’ forms of abuse that they had previously acknowledged [50]. This suggests that recollection of psychological abuse months after it occurred may be less accurate than that of physical or sexual abuse.

*Victim’s profile.* The main demographic and socioeconomic characteristics of the women in our sample are set out in detail in S1 Appendix (Table S2) distinguishing between those who experienced at least one instance of abuse (V) and those who did not (NV). The former were, on average, younger (37.7% less than 45 years old compared with 50.4% for non-abused women), disproportionately of foreign citizenship at birth (18.3% against 4.4%), almost equally educated (i.e., the share with tertiary education is practically the same for the two groups), less likely to have no child living in the household (48.9% against 58.2%), significantly more likely to be unemployed or out of the labour force (39.7% against 23.2%) as well as more likely to live in a household that experienced an economic shock due to job loss for at least one partner during lockdowns (46.6% against 31.0%). Additionally, 28.26% of the victims had lost their job against 21.67% of the non-victims. On average, the partners of abused women were slightly less educated, more likely to be unemployed or non-active and to have lost their jobs during the lockdown.

Typical household characteristics also differed between the two groups of women. Abused women more often reported that it was somewhat difficult or very difficult for the household to make ends meet from one paycheck to another (48.9% against 25.5% for non-abused women). Furthermore, where they lived was more crowded and less likely to have outdoor space. The dummy indicator we constructed to capture crowded living space and lack of privacy, takes value 1 if the household (i) has no outdoor space and (ii) accommodates more than one person per room (excluding bathroom and storage rooms and counting children as half persons). On average, abused women scored higher than non-abused women on this indicator (0.17 against 0.12).

On the whole, this evidence yields a profile for abused women which is broadly familiar in the literature and which we used to define and select the variables for our estimations.

## 5. Empirical strategy

To address the first question, we deployed descriptive statistics, while we resorted to regression analysis for the second and third questions. Our dependent variables in regression analysis are prevalence and fuzzy IPV outcomes (prevalence or fuzzy IPV for short), further distinguished by types of violence. The explanatory variables of interest are our three shocks: job loss following lockdown for at least one partner, partners living with children under 18 during lockdowns, and partners living in crowded space with no privacy during lockdowns. All the three variables are binary.

The list of controls reflects the victim’s profile and includes her age class and citizenship, his and her educational level and employment status, and the household’s economic condition, all measured at or around the time of the interview. We also added two control variables obtained from administrative data: a composite indicator of high-risk alcohol consumption for 2017 in the health district serving the household [74], and the size of the municipality of residence in 2020 [75] (See Table S4 in S2 Appendix). The former serves as proxy for a variable on alcohol abuse (by the partner) that the PANGEA questionnaire did not collect due to the optimal duration limit imposed on CATI interviews; the latter proxies differences in institutional context that may influence the availability of organized support despite the pandemic, e.g., online support from help centres or through pharmacies, hospitals, neighbourhood police etc.

In the IPV literature, endogeneity understood as reverse causation does not emerge as a serious concern for demographic controls like age, education or nationality for the woman or her partner. Similarly, the partner’s employment status may affect his violent behaviour, but the reverse has not been a matter of much investigation. Size of municipality or alcohol consumption in the district where the couple lived are plausibly exogenous with respect to the violent behaviour of the single partner. However, the literature indicates that an important control variable, the woman’s employment status, may suffer from endogeneity [76,77]. Our survey included a question about her employment status at the time of the interview, hence we may interpret our variable as ‘habitual employment status’, which can be affected by IPV. This source of endogeneity may also ‘carry over’ another control variable – ‘household economic conditions’ – to the extent that her earnings contribute to the household budget. For these two variables we adopted a different strategy: a control function and quasi-likelihood estimation to instrument her employment status, a robustness test to verify that removing the economic conditions variable does not significantly affect the estimated impact of our three ‘shocks’ of interest. In what follows we formalize our estimation strategy, including instrumentation, while robustness is discussed in Subsection 6.3.

Let us consider a general model for woman i:


$$\;G\left(s_{i},\;x_{i},\;\beta ,\mathrm{\backslash bold}\gamma \right)=\;\Phi \left(s_{i}\beta +x_{i}\gamma \right)\;$$ (1)

where G(.) is a function such that 0≤G(.)≤1. The vector $s_{i}\;$ includes our pandemic-specific shocks, i.e., our three independent variables of interest, while $x_{i}\;$ comprises control variables. β and γ are the respective coefficient vectors and Φ(.) is the standard normal cumulative distribution function.

The first IPV outcome (*y*_*i*_: prevalence) is estimated by a probit model where $\Phi \left(s_{i}\beta +x_{i}\gamma \right)$ represents the probability of experiencing at least one act of violence.


$$\;P\left(y_{i}=\text{1 }\right|s_{i},x_{i})=\;G\left(s_{i},\;x_{i},\;\beta ,\gamma \right)=\;\Phi \left(s_{i}\beta +x_{i}\gamma \right)$$ (2)

The second IPV outcome ($\overset{*}{\underset{i}{y}})$ is a fuzzy IPV index which measures the experience of violence along a continuum. However, the index is bounded between 0 and 1 whereby neither β nor γ can be consistently estimated by a linear model. We therefore adopted the solution by [78] of a quasi-maximum likelihood (QML) estimation for a functional form, ensuring that estimates of $E\left(\overset{*}{\underset{i}{y}}|{s_{i},\;x}_{i}\right)$ are bounded (between 0 and 1) without an *ad hoc* transformation of boundary values. [78] first proposed a logistic function but used a probit function in a later study due to its advantages in handling endogenous explanatory variables [79], and we adopted this latter solution for our IPV fuzzy index estimation:


$$\begin{array}{c} \begin{array}{c} E\left(\overset{*}{\underset{i}{y}}|{s_{i},\;x}_{i}\right)=\;G\left(s_{i},\;x_{i},\;\beta^{*}\;,\gamma^{*}\right)=\;\Phi \left(s_{i}\beta^{*}+\;x_{i}\gamma^{*}\right)\; \end{array} \end{array}$$ (3)

For both equations 2 and 3 we estimated three specifications: for total IPV, psychological IPV, and physical and/or sexual IPV.

*Tackling endogeneity.* We instrumented her employment status – our endogenous explanatory variable (EEV) – with a combination of two instruments. The first is the female employment rate at municipal level before the pandemic (2019); the second is the variation (difference) in that rate since 2011. The validity and strength of these instruments are discussed in Subsection 5.1. Instruments’ validity and strength). Given that our dependent variables are either binary (prevalence) or fractional (IPV) we resorted to the two-step control function (CF) approach combined with a quasi-likelihood method to carry out instrumentation without falling into the forbidden regression trap [80].

In the prevalence and fuzzy IPV specifications, the CF first stage involves modelling the binary EEV (${Employment}_{i\text{2}})$ as follows:


$$\begin{array}{c} \begin{array}{c} {Employment}_{i\text{2}}={\text{1}[z}_{i}\delta_{2}+\;s_{i}\beta_{2}+\;x_{i}\gamma_{2}+v_{i\text{2}}\geq \text{0}] \end{array} \end{array}$$ (4)

where $v_{i\text{2}}$ is the error term such that $v_{i\text{2}}$|$z_{i}$ ~ Normal (0,1), and **z** is a vector of instruments assumed to be independent of **ν_i2_**.

The CF second stage for IPV prevalence is a probit model that includes the generalized residual ${\hat{r}}_{i}$ obtained from the first stage:


$$\begin{array}{c} \begin{array}{c} P\left(y_{i\text{1}\;}=\text{1}\;\right|\;s_{i},x_{i}{,Employment}_{i},r_{i})=\;\Phi \left({Employment}_{i\text{2}}\tau_{\text{1}}+\;s_{i}\beta_{1}+x_{i}\gamma_{1}+{\hat{r}}_{i\;}\theta_{\text{1}}\right)\; \end{array} \end{array}$$ (5)

where $H_{\text{0}}:\;\theta_{\text{1}}=\text{0}$ tests the null hypothesis that ${Employment}_{i\text{2}}$ has actually been made exogenous.

In the fuzzy IPV estimation, the second stage is a fractional probit model that includes the generalized residual ${\hat{r}}_{i\;}\;$obtained from the first stage:


$$\begin{array}{c} \begin{array}{c} \;E\left(\overset{*}{\underset{i\text{1}}{y}}\;\right|\;s_{i},x_{i,}{Employment}_{i},r_{i\;})=\;\Phi \left({Employment}_{i\text{2}}\overset{*}{\underset{\text{1}}{\tau }}+\;s_{i}\overset{*}{\underset{1}{\beta }}+x_{i}\overset{*}{\underset{1}{\gamma }}+{\hat{r}}_{i\;}\overset{*}{\underset{\text{1}}{\theta }}\right)\; \end{array} \end{array}$$ (6)

where the (non) significance of $\overset{*}{\underset{\text{1}}{\theta }}\;$also indicates that ${Employment}_{i}$ is actually exogenous. For discrete EEV, ${\hat{r}}_{i}$ can correct for endogeneity, particularly when the extent of endogeneity, as measured by  θ, is “small” [82]. Following [81,82], we bootstrapped both stages to obtain valid standard errors for CF estimators. Note finally that the assumption that ${\hat{r}}_{i}$ in both (5) and (6) is a sufficient correction is supported by the finding that  θ is not statistically different from zero in either equation (see Section 6).

### 5.1. Instruments’ validity and strength

Given heterogeneous development patterns at the territorial level, and the related occupational segregation, the two instruments we chose reflect local employment opportunities for women in the long and medium run. As such, they are likely to affect a woman’s decision to work, whereas the reverse effect is negligible if only for statistical reasons. Earlier empirical literature has used proxies of area-specific employment opportunities as instruments for women’s labour market outcomes [76,77,83,84]. In principle, local-level indicators like the average employment rate in a given area may also be affected by the kind of gender norms that influence violence in the area. This may seem to contradict the exclusion restriction assumption that a valid instrument requires. However, we are comparing average employment rates within a geographically small and relatively culturally homogeneous area – Tuscany – whereby the extent to which women work in, say, municipality A compared to municipality B is unlikely to reflect significant differences in gender norms between these municipalities.

The results of first stage estimation (Equation 4) are reported in Table S5 of S2 Appendix and provide evidence of the relevance and strength of our combination of instruments. The female employment rate has a negative and significant association with non-employed women, and a negative and non-significant relation with the variation in female employment rates. Thus, both instrumental variables exhibit the expected signs, though only the first shows strong predictive power. Regarding the exclusion restrictions, neither instrument is significantly correlated with our dependent variables (see Table S6 in S2 Appendix). In the same Appendix we further document the strength of our joint instrument set (Table S7) [85].

## 6. Results and discussion

### 6.1. Main results

*Did IPV worsen?* In our questionnaire, psychological violence included items like forbidding the woman to go to the doctor or hospital, which we termed COVID-specific and listed separately (see Table S1). For each non-COVID-specific item, victims were asked whether they had been abused ‘more’ or ‘less’ compared to before the pandemic, or no change had occurred. The ‘more than before’ category includes three possibilities: abuse occurring for the first time, occurring more frequently or occurring more forcefully. Only a small minority of abused women answered ‘less than before’, the percentage ranging from a tiny 3.1% for sexual abuse to 7.3% for psychological abuse. Depending on the type of violence, slightly more or slightly less than half abused women indicated no change (Table 2). Hence, nearly half the women reporting sexual abuse and 37–38% of those reporting psychological or physical violence answered ‘more than before’. This is clear, if subjective, evidence of heightened violence.

**Table 2:** Percentage distribution of perceived change in IPV experience by violence type.

	Psychological violence	Physical violence	Sexual violence
More than before	37.66	37.05	48.55
Like before	55.05	56.13	48.33
Less than before	7.29	6.81	3.12
All	100.0	100.0	100.0
Total number of victims	141	56	34

Notes: Outcomes with sampling weights and imputed values. Respondents are all women who reported at least one non-COVID-specific abuse during lockdowns.

*The role of shocks.* Table 3 answers our second and third research questions. The table sets out the estimated, average marginal effects of the COVID shocks as well as of the instrumented control variable (women’s employment status). It also reports estimates of the coefficient of generalized residuals in the CF model. From a methodological perspective, the salient finding is that the coefficients of generalized residuals lack significance, which indicates that endogeneity has been controlled for. An additional indication in the same direction is that the CF estimates for the most significant COVID shock (presence of children and job loss) are close to those from probit/fractional regressions (across IPV measures and types). In fact, instrumentation affects the results mainly by reducing the statistical significance of the instrumented variable. This last finding is not new in the literature [77,84,86], and since our interest in the actual role of women’s employment is primarily instrumental, we refer readers to the vast literature on the topic.

**Table 3:** Main estimates.

*A Dependent variable: 1 if victims of IPV.*						
*Model*	Any type of IPV		Psychological IPV		Sexual/physical IPV	
	(1a)	(2a)	(1b)	(2b)	(1c)	(2c)
	Probit	CF	Probit	CF	Probit	CF
*Average predicted value of dependent variable*	0.057	0.057	0.054	0.054	0.025	0.025
	(0.005)	(0.005)	(0.004)	(0.005)	(0.003)	(0.005)
*Average Marginal effects*						
At least one partner lost job (= 1)	0.028***	0.028***	0.025**	0.025**	0.010	0.010
	(0.011)	(0.011)	(0.010)	(0.010)	(0.007)	(0.007)
Children (<18 years) in household (= 1)	0.022**	0.025**	0.024**	0.026***	0.020***	0.023***
	(0.010)	(0.010)	(0.010)	(0.010)	(0.007)	(0.007)
House with no outside space and non-privacy index >= 1 (= 1)	0.014	0.011	0.009	0.006	0.003	0.000
	(0.015)	(0.016)	(0.014)	(0.015)	(0.009)	(0.010)
Woman not working (= 1)	0.034***	0.185	0.036***	0.191	0.019**	0.410
	(0.012)	(0.213)	(0.012)	(0.220)	(0.008)	(0.340)
Generalized residuals		−0.010		−0.050		−0.061*
		(0.009)		(0.053)		(0.035)
Number of observations	2,061	2,061	2,061	2,061	2,061	2,061
B. Dependent variable: fuzzy indicators of IPV.						
**Model**	**Any type of IPV**		**Psychological IPV**		**Sexual/physical IPV**	
	**(1a)**	**(2a)**	**(1b)**	**(2b)**	**(1c)**	**(2c)**
	**Frac.**	**CF**	**Frac.**	**CF**	**Frac.**	**CF**
*Average predicted value of dependent variable*	0.011	0.011	0.015	0.015	0.007	0.007
	(0.001)	(0.001)	(0.001)	(0.002)	(0.001)	(0.001)
*Average marginal effects*						
At least one partner lost job (= 1)	0.002	0.002	0.002	0.003	0.001	0.002
	(0.002)	(0.002)	(0.003)	(0.003)	(0.002)	(0.002)
Children (<18 years) in household (= 1)	0.013***	0.014***	0.016***	0.018***	0.009***	0.010***
	(0.003)	(0.003)	(0.004)	(0.004)	(0.002)	(0.003)
House with no outside space and non-privacy index >= 1 (= 1)	0.006	0.005	0.007	0.006	0.005	0.004
	(0.004)	(0.004)	(0.005)	(0.005)	(0.003)	(0.004)
Woman not working (= 1)	0.007**	0.093	0.011***	0.133	0.004*	0.051
	(0.003)	(0.124)	(0.004)	(0.165)	(0.002)	(0.088)
Generalized residuals		−0.016		−0.023	0.001	−0.010
		(0.012)		(0.016)		(0.009)
Number of observations	2,061	2,061	2,061	2,061	2,061	2,061

Notes: *** p < 0.01, ** p < 0.05, * p < 0.1. Outcomes are average marginal effects. Standard errors in brackets. Women not working are women who are not employed or self-employed in paid activities. In columns (2a), (2b) and (2c) the instruments are female employment rate at municipal level and its variation with respect to 2011. The bootstrapped standard errors were obtained through 500 replicated weights. Frac = fractional. CF = Control-function method. Control variables included but not shown in the table are her age, nationality, his and her educational levels and employment status, economic condition of household, the size of municipality of residence, and high-risk alcohol consumption in health district serving the household.

In reading the table recall that, unlike the CF model, the probit and the fractional models do not control for endogeneity and are used when the dependent variable is measured by prevalence and by the fuzzy index, respectively. From a substantive perspective, the findings reveal a hierarchy of the COVID-specific effects. The presence of children under 18 capturing parental overburden yields the most consistently significant and among the largest effects across different models, measurement and types of IPV. For example, with reference to the CF model estimates, the increase in prevalence amounts to 2.5 pp. for all types of abuse, i.e., a 44% rise with respect to the average predicted value (column 2a, Table 3). Psychological violence is the strongest driver in this case (+2.6 pp.) though the difference with respect to sexual and/or physical violence is small. The results for the fuzzy measure of violence tell a similar story: the estimated increase in the IPV index for all types of violence amounts to 0.014 index points in the CF model, corresponding to a 127% rise with respect to the average predicted value. In this case too, the increase is primarily driven by psychological violence.

The results are mixed in the case of job loss, and they vary with IPV measurement. The estimated effect is almost as strong as for parental overburden when IPV is measured by prevalence: + 2.8 pp. for violence of all types corresponding to a 49% rise. In this case too, the aggregate result is driven by psychological IPV (+2.5 pp.) while the effect for physical/sexual IPV is lower and lacks significance (+1.0 pp). In comparison, significance drops below conventional levels across models and violence types when IPV is measured by the fuzzy index. Finally, confinement in crowded space apparently had no significant effect across models, measures and violence types, although the sign is as expected.

Evidence from the qualitative in-depth interviews of the PANGEA project [50] may throw light on the above results. Some of the victims taking part in qualitative interviews linked their experience of violence during lockdowns with the conflict arising from the need to constantly renegotiate a childcare workload that had become more onerous and complex during confinement. An additional source of conflict they highlighted was the fact that children were perceived by some partners as vehicles of contagion [50].

In contrast, most victims viewed confinement in spaces lacking privacy as an impediment to organizing support from outside rather than a factor conducive to violence. Controlling behaviour was widespread during confinement and these women felt that partners could check on them everywhere, even in the bathroom, no matter how large the living space might have been.

All this may offer some explanation of why parental overburden and confinement in crowded space lacking privacy appear to have had the strongest and the weakest impacts, respectively. In between stands job loss for either partner, which may be viewed as epitomizing COVID-specific economic shocks. An important distinction that would have been lost had we only used prevalence to measure IPV, is that job loss appears to have played a significant role only for less severe IPV abuses. This is indicated by the fact that (i) the increase in overall prevalence was driven by psychological rather than physical or sexual violence, and (ii) when severity of IPV is accounted for (by our fuzzy index) the association with job loss consistently loses significance.

Concerning controls (other than her employment status), and focusing again on the CF estimates, the results for foreign status, young age and education are worth mentioning. The marginal effect of being of foreign citizenship, typically of migrant origin from outside but also from within the EU, is consistently significant across all estimates and of an order of magnitude larger than for any other variable. For example, the average, predicted likelihood of reporting any kind of violence nearly triples for foreign citizens. In comparison, estimated marginal effects are lower for being young (less than 31 years) and living with a low educated partner, although both these variables emerge as significant risk factors across estimates. In all these cases measurement matters since both the level of significance and the size of the marginal effects tend to be higher when severity is accounted for, i.e., for fuzzy outcomes rather than prevalence. Interestingly, her education seems to have been a protective factor since marginal effects for this variable carry a negative sign, although exhibiting weaker significance than for partner’s education in several instances.

Size of municipality, partner’s employment status, and alcohol consumption in the health district of residence rarely or never exhibit statistical significance. In the case of alcohol consumption this may seem surprising given clear indications to the contrary in the literature, but we cannot rule out that the proxy we used for individual alcohol abuse is poor. Also, confinement need not have increased alcohol consumption for all users: as some of the women participating in the qualitative survey pointed out, certain users may have found it more difficult to secure supply during lockdown [50]. Finally, the findings are mixed for the making-ends-meet variable which is discussed in greater depth in the following paragraphs.

### 6.2. Gender norms as a moderation factor

Was the effect of parental overburden moderated by gender-related attitudes? There is a fair deal of consensus in the literature that authoritarian parenting and resistance to sharing childcare are salient indicators of traditional gender attitudes, especially on the part of the male partner. Adherence to these attitudes may thus have affected the impact of parental overburden on IPV during the pandemic. The PANGEA survey did not directly investigate partners’ views; hence we proxied gender-related attitudes with two of our most impactful control variables, namely male partner’s education and woman’s citizenship. Low education has long been associated with traditional gender values [87], and its moderating role in the occurrence of violence is especially worth investigating for the male partner. Concerning foreign status, our survey only recorded the woman’s citizenship, and we know that the majority of abused women in the survey originated from countries that rank low on international indexes of gender equality (like Honduras, Peru, Afghanistan, Morocco among others) or from former socialist countries where women’s employment tends to be accepted but support for women’s equality remains comparatively low [88]. This evidence is compatible with the possibility that, in comparison to local women, women of foreign citizenship felt or were actually less empowered to diffuse tensions over parenting.

The estimations in Table 3 were thus repeated after adding two terms, respectively the interaction of parental overburden with the woman’s foreign status and with the partner’s education. The first notable result is that our main estimates (the average marginal effects of the COVID shocks reported by Table 3) hardly change with the introduction of the interaction terms. Moreover, both these terms attain statistical significance across specifications for fuzzy IPV outcomes but hardly ever for prevalence IPV, echoing our previous findings on how measurement influences outcomes (Section 6.1). Finally, we gauged the strength of the interaction from the differential marginal effect of parental overburden for women of foreign citizenship compared to local women and for women with low-to-middle educated partner compared to women with a highly educated partner. Both these differential effects are significant at conventional levels for fuzzy IPV outcomes, not for prevalence IPV; also, the differential effect is larger for foreign citizenship (Table 4).

**Table 4:** Moderation of parental overburden effects by foreign status and partner’s education.

	Panel A: 1 if victims of IPV			Panel B: fuzzy indicators of IPV		
	Any IPV	Psychological	Sexual/physical	Any IPV	Psychological	Sexual/physical
Interactions with Children	Control Function			Control Function		
*Average Marginal effects*						
Italian	0.021 (0.011)	0.022 (0.010)	0.017 (0.007)	0.010 (0.003)	0.013 (0.004)	0.007 (0.002)
Foreign	0.118 (0.064)	0.119 (0.063)	0.133 (0.050)	0.078 (0.024)	0.099 (0.030)	0.058 (0.021)
**Diff.**	**0.097** **(0.065)**	**0.097** **(0.064)**	0.116** **(0.051)**	0.068*** **(0.024)**	0.086*** **(0.031)**	0.051** **(0.022)**
*Average Marginal effects*						
High-educ. partner	0.019 (0.011)	0.020 (0.011)	0.017 (0.007)	0.008 (0.002)	0.012 (0.003)	0.005 (0.002)
Low/mid-educ. partner	0.045 (0.023)	0.048 (0.023)	0.045 (0.021)	0.029 (0.009)	0.035 (0.011)	0.023 (0.008)
**Diff.**	**0.025** **(0.026)**	**0.028** **(0.025)**	**0.028** **(0.022)**	0.020** **(0.009)**	0.023** **(0.011)**	0.017** **(0.009)**

Notes: Average marginal effects of interactions with parental overburden (presence of children under 18). Both interactions (foreign status × children; partner’s education × children) are included in the same model simultaneously. Standard errors in parentheses. Diff. = AME for group 1 − AME for group 0; SE computed from the variance-covariance matrix of the marginal effects. Statistical significance stars are reported only for the difference estimates

(Diff. rows). *** p < 0.01, ** p < 0.05, * p < 0.10. Only the control function estimates are reported. The corresponding probit and fractional estimates yield broadly similar conclusions. The full table is available upon request.

A plausible indication we would draw from the above findings is that adherence to traditional gender norms on the part of one or both partners may have exacerbated the effect of parental overburden on the intensity and severity of violence rather than on prevalence. However, our use of proxy variables to obtain these results warrants caution in interpreting them.

### 6.3. Sensitivity and robustness

We carried out a number of tests in order to assess the robustness and sensitivity of our key findings. The first robustness test involved repeating the estimation after dropping the making-ends-meet variable (Table S8, S3 Appendix). This variable gauges the prevalent condition of the household in late 2021, when the economy (GDP) had almost recovered to pre-pandemic levels; as such, it need not have been influenced by job loss during lockdowns. However, it probably captured ‘usual’ economic conditions for that household, since poverty tends to persist in time. Removing this variable serves two purposes, namely verifying that the effects of interest are not affected by endogeneity, and that our third COVID-specific shock, crowding and lack of privacy, does not act as a proxy for poor economic conditions. The results are reassuring since the effects of our ‘shocks’ maintain the same level of statistical significance and very similar values after removing this variable.

Redistributing sampling weights in the third round of our data collection in order to preserve abuse prevalence from previous rounds could have distorted the results. In our second robustness test (Table S9, S3 Appendix) we therefore repeated all estimations after dropping all the observations from the third round. In comparison with our main estimates (Table 3), all marginal effects of parental overburden on prevalence IPV get smaller in size and lack significance across models and violence types in the reduced sample, whereas job loss effects gain both in size and significance across the board. However, results are very different for the marginal effects on fuzzy IPV outcomes. In this case job loss effects lose significance across the board compared to the main estimates (although most gain in magnitude) while those for parental overburden reduce in size but retain significance for aggregate (all types) as well as psychological IPV. Confinement in crowded space continues to exhibit nonsignificant effects.

Arguably, these results are consistent with our main findings in one important respect, namely that measurement by prevalence may lead to overstating the role of factors involved in widespread types of violence while understating the role of factors influencing intensity and/or severity of violence. We therefore see no strong reason to question the hierarchy of effects that can be inferred from the main estimates.

As noted earlier (Sections 3 and 4) we found a lower, overall IPV prevalence figure than expected when designing the PANGEA survey. The small number of IPV cases on which our results rest may thus raise concerns about the statistical power of our analysis. While ex-post power analysis is not advisable, comparing Minimum Detectable Effects (MDEs henceforth) with actual estimates reveals possible power deficiencies [89–91]. We therefore compared all our average estimated marginal effects (AMEs) for the three covariates of interest with the corresponding MDEs computed at 0.80 power and three different significance levels (0.01, 0.05, and 0.10: Table S10, S3 Appendix) The comparison suggests that, at conventional levels (0.80 for power, 0.05 for significance) our estimates of the impact of parental overburden on all types of fuzzy IPV outcomes and on sexual and physical IPV prevalence are adequately powered. In contrast, the estimated AMEs of job loss or confinement in crowded space appear to be underpowered across models and specifications (although it is worth recalling that some exhibit conventional statistical significance, specifically the AMEs of job loss on prevalence IPV). We cannot therefore rule out that small sample size contributed to lack of significance for the effects of confinement in crowded space or to the patchy significance pattern of the job loss effects.

Finally, we carried out sensitivity tests by probing the assumptions about errors in the CF model (see Table S11 and Table S12). The results are reassuring, but we relegate the evidence and the related discussion to S3 Appendix for reasons of length.

## 7. Conclusion and limitations

As the dust settled after the upheaval of the COVID-19 pandemic, we revisited the implications for IPV, an issue that received enormous attention when the pandemic was still raging but may still be worth analysing. We were in fact able to leverage the information collected for a mixed-method research project that began more than a year after the pandemic started and soon after the last strict confinement measure was removed. In this article, we exploited the project’s quantitative survey while also using evidence from the qualitative interviews in order to interpret the findings.

To our knowledge the data source we built and exploited is the only representative survey investigating IPV in Italy during the pandemic, and even if it is representative of a (large) region rather than the whole country, it brings to the literature evidence from the first country to be hit by COVID-19 after China, and one of the worst hit. The cross-sectional nature of the survey has imposed limitations on causal investigation. Our attempt here was to at least partially overcome these limitations by exploiting the unexpected timing and nature of some potential triggers of IPV, but we acknowledge that causal inference from our results may be controversial.

Our key findings are as follows:

- Overall, the evidence is in favour of a worsening of IPV during lockdowns in Tuscany, although comparisons before and after the pandemic for this region must be taken with caution. A large minority of the women having reported abuses during lockdowns (more than one third in each type of violence) perceived those abuses to be worse than before and only a handful perceived a lessening of violence.
- We focused our investigation on three specific shocks: job loss following lockdown for one or both partners, parental overburden, and confinement in crowded space lacking privacy during lockdowns. The second emerged as the most consequential across estimation models and IPV outcome measurements, followed by job loss. Confinement in crowded space with no privacy did not emerge as a significant factor.
- Gender equality attitudes may have affected the impact of parental overburden since the interaction between our proxies for traditional attitudes and parental overburden appears to have exacerbated IPV. However, the interpretation of this finding deserves caution given the use of proxies.
- Measurement of IPV outcomes matters. Accounting for severity in addition to prevalence allowed us to discern between the factors that appear to have primarily influenced prevalence (e.g., job loss) and those that also influenced severity (presence of minors). Among control variables, moreover, foreign citizenship, young age and low education of the partner tend to acquire higher significance or exhibit a larger effect when IPV measurement takes account of severity.

The post-estimation tests we conducted broadly confirm our estimation strategy, suggesting that our main results are fairly robust despite the limitations imposed by the data. However, some of our estimates are underpowered, especially the effects of job loss and confinement in crowded space.

The policy implications are non-trivial. For one, IPV worsened non-negligibly as documented by other studies, but the worsening was apparently greater for sexual violence, unlike what some of these studies reported (Section 2). This not only calls for the design of preventative measures, but also for targeting them to the women more at risk of sexual abuse in pandemic-like situations. For another, both the literature and policy action de facto prioritized economic shocks over other shocks, those concerning parenting in particular. Only a few countries made an effort to keep schools open while measures to counter economic distress were put in place. School closure may have been justified by the risk of contagion, but knowledge that this measure not only hinders learning but also significantly raises the risk of violence among couples with minors warrants some reconsideration of priorities and group targeting in pandemic times. All the more so since different types of shocks appear to have impacted IPV types in differentiated manners. By using a comprehensive measure of IPV outcomes, we specifically found that economic shocks are more consequential for psychological than for other types of abuses. While more evidence on differential impact by abuse type is needed to exclude that our finding reflects specific local conditions, or to conclude that it can be generalized to non-pandemic times, the more general lesson we feel entitled to draw is that measuring violence comprehensively rather than partially is essential to gain robust evidence for policy guidance.

## Supporting information

S1 Appendix Violence types and acts, severity weights, descriptive statistics, and imputation details. S1-S3 tables.
(DOCX)

S2 Appendix Control variables’ estimates and Instrument diagnostics. S4-S7 tables.
(DOCX)

S3 Appendix Robustness analysis and Sensitivity tests. S8-S12 tables.
(DOCX)
