# Supplementary material for: Intimate partner violence during lockdown in Tuscany, Italy: Economic or confinement-related shocks?
Source: PLoS One. 2026 Jun 24;21(6):e0349889. doi: 10.1371/journal.pone.0349889 (PMC13293386; doi:10.1371/journal.pone.0349889)
Supplement: S1 Appendix — S1-S3 tables. (DOCX) [file pone.0349889.s001.docx]

## S1 Appendix - Violence types and acts, severity weights, descriptive statistics, and imputation details

The fuzzy index we use here accounts for prevalence and severity, not for intensity. This is because intensity was investigated ordinally rather than cardinally (‘more than before’, ‘like before’, ‘less than before’), and only for non-COVID-specific abuses (see Section 5). We adapted the method of [19] using the average number of distinct violent acts experienced by each woman rather than the frequency of individual acts, naming this average as “intensity”.

Table S1 - Violence types and acts, and severity weights

| Violence Types and Acts | Severity weights (type specific) |
| --- | --- |
| *Psychological violence (COVID and non-COVID specific)*  During the lockdown periods starting in March 2020, did your partner… |  |
| Forbid or try to prevent you to go to the doctor, to the hospital, or other health care places?^1^ | 1.253 |
| Restrict or try to restrict your contact with family of origin, relatives, or friends?^2^ | 0.827 |
| Forbid or try to prevent you to work outside the home? | 1.117 |
| Forbid or try to prevent you to leave the house?^1^ | 1.253 |
| Question your fidelity? | 0.732 |
| Belittle or humiliate you in private? | 0.603 |
| Belittle or humiliate you in public? | 0.724 |
| Prevent you from making your own decisions about family finances and purchases? | 0.836 |
| Do anything to intentionally frighten or intimidate you, such as yelling and destroying objects? | 0.738 |
| Threaten to take the children away from you? | 1.183 |
| Hurt or threaten to harm your children?^3^ | 1.345 |
| Hurt or threaten to hurt a person close to you? | 1.327 |
| Threaten to hurt you? | 1.062 |
| *Physical violence*  During the lockdown periods starting in March 2020, did it happen that your partner … |  |
| Slap you? | 0.832 |
| Hit you with a fist or a contusive object, or bite or kick you? | 0.985 |
| Throw anything at you or an object that hurt you or otherwise could have hurt you? | 0.951 |
| Grab you, turn your arm or pull your hair, hurting or frightening you? | 0.919 |
| Push or pull you, hurting or frightening you? | 0.748 |
| Intentionally try to strangle or choke you? | 1.146 |
| Intentionally try to burn you? | 1.161 |
| Use a gun, knife, other weapon, or other dangerous substances against you? | 1.161 |
| Beat your head against something? | 1.097 |
| *Sexual violence*  During the lockdown periods starting in March 2020, did it happen that your partner … |  |
| Force you into sexual intercourse by threatening, holding you down or hurting you in some way? | 1.054 |
| Attempt to force you into sexual intercourse by threatening you, holding you down, or doing hurting you any other way? | 1.029 |
| Make you take part in sexual activities against your will or in a situation in which you were unable to refuse? | 0.981 |
| Did you happen to consent to sexual activity, even though you didn't feel like it, because you were afraid of what your partner would do if you refused? | 0.936 |

Notes: Severity weights extracted and adapted from [19]. The weights are normalized such that their mean within each violence type is equal to one. 1. Weights considered to be equivalent to the item “Forbidden you to leave the house, taken away your car keys or locked you up?”; 2. Weight considered to be equivalent to the average between the weight of the items “Tried to keep you from seeing your friends?” and “Tried to restrict your contact with your family of birth or relatives?”; 3. Weight considered to be equivalent to the average between the weight of the items “Hurt your children?” and “Threatened to hurt your children?”.

Table S2 – Sample demographic and socioeconomic characteristics

|  |  | NV |  | V |
| --- | --- | --- | --- | --- |
| Variable | Category | % |  | % |
| Age group (years) | 18-34 | 15.86 |  | 20.91 |
|  | 35-44 | 21.82 |  | 29.53 |
|  | 45-54 | 35.93 |  | 30.97 |
|  | 55-64 | 26.39 |  | 18.59 |
| Nationality group | Italian | 95.61 |  | 81.74 |
|  | Foreigner | 4.39 |  | 18.26 |
| Education background | Lower secondary education or less | 18.25 |  | 21.90 |
|  | Upper secondary education | 46.81 |  | 44.18 |
|  | Higher education or more | 34.95 |  | 33.91 |
| Education background of partner | Lower secondary education or less | 25.9 |  | 26.55 |
|  | Upper secondary education | 46.8 |  | 46.64 |
|  | Higher education or more | 27.3 |  | 26.81 |
| Employment status | Employed | 76.82 |  | 60.31 |
|  | Unemployed | 5.44 |  | 18.11 |
|  | Outside labour force | 17.74 |  | 21.58 |
| Employment status of partner | Employed | 86.1 |  | 81.7 |
|  | Unemployed | 1.44 |  | 9.31 |
|  | Outside labour force | 12.47 |  | 9.03 |
| At least one partner lost job | No | 69.00 |  | 53.39 |
|  | Yes | 31.00 |  | 46.61 |
| Make ends meet at the end of the month | Easy enough to very easy | 74.54 |  | 51.11 |
|  | Some difficulty to very difficult | 25.46 |  | 48.89 |
| Number of children in the household | 0 | 58.20 |  | 48.89 |
|  | 1 | 23.2 |  | 28.30 |
|  | ≥ 2 | 18.65 |  | 22.81 |
| Household members (n) | 2 | 27.2 |  | 24.29 |
|  | ≥ 3 | 72.79 |  | 75.71 |
| Rooms (n) | ≤ 2 | 4.05 |  | 7.87 |
|  | ≥ 3 | 95.95 |  | 92.13 |
| House with and without outside space | With outside space | 38.36 |  | 26.52 |
|  | No outside space | 61.64 |  | 73.48 |
| Municipality population | 0-49,999 | 48.46 |  | 56.98 |
|  | 50,000-99,000 | 30.25 |  | 24.22 |
|  | 100,000 or more | 21.29 |  | 18.80 |

Note: Outcomes with sampling weights and imputed values. NV refers to women that did not experience IPV; V refers to women that experienced IPV.

*Imputation details.* For the imputation of missing values for non-violence-related variables, we employed a univariate stochastic approach using logistic regression, ordered logistic regression, and multinomial logistic regression, depending on the type of the variable to be imputed. By fitting regression models to the observed data, the method predicts the most likely category for each missing observation, based on patterns in the covariates. This approach assumes that the missingness is related to these covariates and that the imputed values can be reliably inferred from the observed relationships.

For the imputation of missing values for variables of violent acts, we implemented a second method using random probability-matching approach, designed to match the observed probability distributions of the data. In this method, a random variable was generated for each case. We then assigned imputed values by comparing these random numbers against the probability distributions derived from the observed data. This approach aims to maintain the distributional properties of the dataset by ensuring that the imputed values reflect the underlying patterns in the observed data.

In our final sample, we imputed 23 values across four variables of socioeconomic characteristics from 21 observations, and 256 values across 26 variables of violent acts from 192 observations. Table S3 shows the number and proportion of imputed values for variables with missing responses. The proportion of non-responses is consistently below 1%.

Table S3 – Imputation details

| Variables | # Imputed | % Imputed |
| --- | --- | --- |
| *Non-violence-related variables* |  |  |
| Education level | 1 | 0.05% |
| Employment status | 3 | 0.15% |
| Partner employment status | 11 | 0.53% |
| Number of rooms | 8 | 0.39% |
| *Variables of Violent acts* |  |  |
| *Psychological* |  |  |
| Forbid or try to prevent you to go to the doctor, to the hospital, or other health care places? | 9 | 0.44% |
| Restrict or try to restrict your contact with family of origin, relatives, or friends? | 10 | 0.49% |
| Forbid or try to prevent you to work outside the home? | 11 | 0.53% |
| Forbid or try to prevent you to leave the house?^1^ | 9 | 0.44% |
| Question your fidelity? | 10 | 0.49% |
| Belittle or humiliated you in private? | 10 | 0.49% |
| Belittled or humiliated you in public? | 12 | 0.58% |
| Prevent you from making your own decisions about family finances and purchases? | 10 | 0.49% |
| Do anything to intentionally frighten or intimidate you, such as yelling and destroying objects? | 11 | 0.53% |
| Threat to take the children away from you? | 5 | 0.24% |
| Hurt or threat to harm your children? | 2 | 0.10% |
| Hurt or threat to hurt a person close to you? | 10 | 0.49% |
| Threat to hurt you? | 9 | 0.44% |
| *Physical* |  |  |
| During the lockdown periods starting in March 2020, did it happen that your partner … |  |  |
| Slap you? | 10 | 0.49% |
| Hit you with a fist or a contusive object, or bite or kick you? | 9 | 0.44% |
| Throw anything at you or an object that hurt you or otherwise could have hurt you? | 10 | 0.49% |
| Grab you, turn your arm or pulled your hair, hurting or frightening you? | 11 | 0.53% |
| Push or pull you, hurting or frightening you? | 11 | 0.53% |
| Intentionally try to strangle or choke you? | 13 | 0.63% |
| Intentionally try to burn you? | 11 | 0.53% |
| Use a gun, knife, other weapon, or other dangerous substances against you? | 9 | 0.44% |
| Beat your head against something? | 9 | 0.44% |
| *Sexual* |  |  |
| During the lockdown periods starting in March 2020, did it happen that your partner … |  |  |
| Force you into sexual intercourse by threatening, holding you down or hurting you in some way? | 10 | 0.49% |
| Attempt to force you into sexual intercourse by threatening you, holding you down, or hurting you any other way? | 11 | 0.53% |
| Make you take part in sexual activities against your will or in a situation in which you were unable to refuse? | 11 | 0.53% |
| Did you happen to consent to sexual activity, even though you didn't feel like it, because you were afraid of what your partner would do if you refused? | 13 | 0.63% |
