# Supplementary material for: Intimate partner violence during lockdown in Tuscany, Italy: Economic or confinement-related shocks?
Source: PLoS One. 2026 Jun 24;21(6):e0349889. doi: 10.1371/journal.pone.0349889 (PMC13293386; doi:10.1371/journal.pone.0349889)
Supplement: S2 Appendix — S4-S7 tables. (DOCX) [file pone.0349889.s002.docx]

## S2 Appendix – Control variables’ estimates and Instrument diagnostics

Table S4 - Control variables’ estimates

A. Dependent variable: 1 if victims of IPV

|  | Any type of IPV | | Psychological IPV | | Sexual and/or physical IPV | |
| --- | --- | --- | --- | --- | --- | --- |
|  | (1a) | (2a) | (1b) | (2b) | (1c) | (2c) |
| *Model* | Probit | CF | Probit | CF | Probit | CF |
| *Average predicted value of dependent variable* | 0.057 | 0.057 | 0.054 | 0.054 | 0.025 | 0.025 |
|  | (0.005) | (0.005) | (0.004) | (0.005) | (0.003) | (0.005) |
| *Average Marginal effect* |  |  |  |  |  |  |
| Partner not working (=1) | 0.009 | -0.009 | 0.009 | -0.009 | 0.012 | -0.010 |
|  | (0.014) | (0.022) | (0.014) | (0.022) | (0.010) | (0.012) |
| Difficult to make ends meet (=1) | 0.040*** | 0.025 | 0.044*** | 0.028 | 0.029*** | 0.011 |
|  | (0.012) | (0.019) | (0.012) | (0.019) | (0.008) | (0.011) |
| Municipality < 50k pop. (=1) | 0.014 | 0.009 | 0.012 | 0.007 | 0.009 | 0.003 |
|  | (0.009) | (0.011) | (0.009) | (0.010) | (0.006) | (0.008) |
| Foreigner (=1) | 0.099*** | 0.097*** | 0.099*** | 0.098*** | 0.080*** | 0.078*** |
|  | (0.029) | (0.030) | (0.029) | (0.030) | (0.022) | (0.024) |
| Low education (=1) | -0.017 | -0.035* | -0.014 | -0.032* | -0.013** | -0.033*** |
|  | (0.011) | (0.019) | (0.010) | (0.019) | (0.006) | (0.013) |
| Partner has low education (=1) | 0.020* | 0.023* | 0.019* | 0.022* | 0.023*** | 0.026*** |
|  | (0.012) | (0.013) | (0.011) | (0.012) | (0.008) | (0.010) |
| Age <= 31 years old (= 1) | 0.053** | 0.048* | 0.058** | 0.052** | 0.028* | 0.020 |
|  | (0.025) | (0.025) | (0.026) | (0.026) | (0.015) | (0.015) |
| High-risk alcohol consumption | 0.002 | 0.002 | 0.002 | 0.002 | 0.001 | 0.001 |
|  | (0.001) | (0.001) | (0.001) | (0.001) | (0.001) | (0.001) |
| Number of observations | 2,061 | 2,061 | 2,061 | 2,061 | 2,061 | 2,061 |

B. Dependent variable: fuzzy indicators of IPV

|  | Any type of IPV | | Psychological IPV | | Sexual and/or physical IPV | |
| --- | --- | --- | --- | --- | --- | --- |
|  | (1a) | (2a) | (1b) | (2b) | (1c) | (2c) |
| Model | Frac. | CF | Frac. | CF | Frac. | CF |

| *Average predicted value of dependent variable* | 0.011 | 0.011 | 0.015 | 0.015 | 0.007 | 0.007 |
| --- | --- | --- | --- | --- | --- | --- |
|  | (0.001) | (0.001) | (0.001) | (0.002) | (0.001) | (0.001) |

| *Average marginal effect* |  | |  |  |  | |  |  |
| --- | --- | --- | --- | --- | --- | --- | --- | --- |
| Partner not working (=1) | | 0.008* | 0.001 | 0.009* | -0.000 | 0.007** | | 0.002 |
|  | | (0.004) | (0.005) | (0.006) | (0.007) | (0.003) | | (0.005) |
| Difficult to make ends meet (=1) | | 0.012*** | 0.007* | 0.016*** | 0.009 | 0.008*** | | 0.005* |
|  |  | (0.003) | (0.004) | (0.004) | (0.006) | (0.002) | | (0.003) |
| Municipality < 50k pop. (=1) | | 0.006*** | 0.004* | 0.007** | 0.005 | 0.004*** | | 0.004* |
|  |  | (0.002) | (0.002) | (0.003) | (0.003) | (0.002) | | (0.002) |
| Foreigner (=1) | | 0.039*** | 0.038*** | 0.049*** | 0.048*** | 0.029*** | | 0.028*** |
|  | | (0.010) | (0.011) | (0.013) | (0.013) | (0.007) | | (0.009) |
| Low education (=1) | | -0.004* | -0.010** | -0.005 | -0.013** | -0.004** | | -0.007** |
|  | | (0.002) | (0.005) | (0.003) | (0.006) | (0.002) | | (0.004) |
| Partner has low education (=1) | | 0.012*** | 0.013*** | 0.013*** | 0.014*** | 0.011*** | | 0.011*** |
|  | | (0.004) | (0.004) | (0.004) | (0.005) | (0.003) | | (0.003) |
| Age <= 31 years old (= 1) | | 0.022*** | 0.019** | 0.026*** | 0.022** | 0.017*** | | 0.015** |
|  | | (0.007) | (0.007) | (0.009) | (0.009) | (0.006) | | (0.007) |
| High-risk alcohol consumption | | 0.000 | 0.000 | 0.001 | 0.001 | 0.000 | | 0.000 |
|  | | (0.000) | (0.000) | (0.001) | (0.000) | (0.000) | | (0.000) |
| Number of observations | | 2,061 | 2,061 | 2,061 | 2,061 | 2,061 | | 2,061 |

Notes: *** p<0.01, ** p<0.05, * p<0.1. Outcomes are average marginal effects. Standard errors in brackets. Women not working are women who are not employed or self-employed in paid activities. In columns (2a), (2b) and (2c) the instruments are female employment rate at municipal level and its variation with respect to 2011. The bootstrapped standard errors were obtained through 500 replicated weights. Frac = fractional. CF = Control-function method.

Table S5 - First stage estimations

| Average Marginal effect | Probit First stage |
| --- | --- |
| Children (<18 years old) in the household = 1 | -0.024 |
|  | (0.020) |
| If the house has no outside space and non-privacy index >= 1 | 0.035 |
|  | (0.029) |
| Female employment rate by municipality | 0.184*** |
|  | (0.030) |
| 2019-2011 difference in female employment rate by municipality | -0.024 |
|  | (0.020) |
|  |  |
| Observations | 2,061 |

Notes: *** p<0.01, ** p<0.05, * p<0.1. Outcomes are average marginal effects. Bootstrapped Standard errors are within brackets, obtained through 500 replicated weights.

Table S6 – Correlations between dependent variables and instruments

| Correlation | IPV Fuzzy index | IPV Prevalence | Instrument 1 | Instrument 2 |
| --- | --- | --- | --- | --- |
| IPV Fuzzy index | 1.0000 |  |  |  |
| IPV Prevalence | 0.6868* | 1.0000 |  |  |
| Instrument 1 | 0.0169 | 0.0203 | 1.0000 |  |
| Instrument 2 | -0.0024 | -0.0777 | 0.0654* | 1.0000 |

Notes: * p<0.1. Pairwise correlation type depends on the type of the variables: polychoric (ordinal), tetrachoric (ordinal with two categories), polyserial (continuous and ordinal), biserial (continuous and ordinal with two categories), and Pearson (both with over 10 categories). Instrument 1 refers to Female employment rate by municipality and Instrument 2 to 2019-2011 difference in female employment rate by municipality.

*Instruments’ strength*. To the best of our knowledge, there is no specific test for instrument strength in nonlinear models. Therefore, we employed 2SLS models and report the first-stage F-statistics as per [87]. In this approach, we treat Equations 4 and 5 as linear probability models and consider Equation 6 as an unbounded continuous variable. Table S5 presents the Effective F-statistics for the first stage. The F-statistic indicates a critical value at τ = 5%, which confirms the sufficient joint strength of our instruments.

Table S7 - Montiel-Pflueger robust weak instrument test

| Effective F statistic: 7.953 |  |
| --- | --- |
| Critical Values | 2SLS |
| τ = 5% | 7.011 |
| τ = 10% | 5.245 |
| τ = 20% | 4.223 |
| τ = 30% | 3.843 |
