# Supplementary material for: Intimate partner violence during lockdown in Tuscany, Italy: Economic or confinement-related shocks?
Source: PLoS One. 2026 Jun 24;21(6):e0349889. doi: 10.1371/journal.pone.0349889 (PMC13293386; doi:10.1371/journal.pone.0349889)
Supplement: S3 Appendix — S8-S12 tables. (DOCX) [file pone.0349889.s003.docx]

## S3 Appendix – Robustness analysis and sensitivity tests

*Robustness analysis*

Table S8 – Estimates without make-ends meet variable

1. Dependent variable: 1 if victims of IPV

|  | Any type of IPV | | Any type of IPV | | | |  |
| --- | --- | --- | --- | --- | --- | --- | --- |
|  | (1a) | (2a) | (1b) | | (2b) | |  |
| *Model* | *Probit* | *CF* | *Probit* | | *CF* | |  |
|  | All variables | | Without make-ends meet variable | | | |  |
| *Average Marginal effect* |  |  |  | | |  |  |
| At least one partner lost job (= 1) | 0.028*** | 0.028*** | | 0.035*** | | 0.036*** | |
|  | (0.011) | (0.011) | | (0.011) | | (0.011) | |
| Children (<18 years) in household (= 1) | 0.022** | 0.025** | | 0.025** | | 0.027** | |
|  | (0.010) | (0.010) | | (0.010) | | (0.011) | |
| House with no outside space and non-privacy index >= 1 (= 1) | 0.014 | 0.011 | | 0.017 | | 0.013 | |
|  | (0.015) | (0.016) | | (0.015) | | (0.016) | |
| Woman not working (= 1) | 0.034*** | 0.185 | | 0.042*** | | 0.214 | |
|  | (0.012) | (0.213) | | (0.013) | | (0.248) | |
| Generalized residuals |  | -0.051 | |  | | -0.054 | |
|  |  | (0.054) | |  | (0.059) | | |
| Number of observations | 2,061 | 2,061 | 2,061 | | 2,061 | |  |

1. Dependent variable: fuzzy indicators of IPV

|  | Any type of IPV | | Any type of IPV | |
| --- | --- | --- | --- | --- |
|  | (1a) | (2a) | (1b) | (2b) |
| *Model* | *Fractional* | *CF* | *Fractional* | *CF* |
|  | All variables | | Without make-ends meet variable | |
| *Average Marginal effect* |  |  |  |  |
| At least one partner lost job (= 1) | 0.002 | 0.002 | 0.004 | 0.004 |
|  | (0.002) | (0.002) | (0.003) | (0.002) |
| Children (<18 years) in household (= 1) | 0.013*** | 0.014*** | 0.013*** | 0.014*** |
|  | (0.003) | (0.003) | (0.003) | (0.003) |
| House with no outside space and non-privacy index >= 1 (= 1) | 0.006 | 0.005 | 0.007* | 0.005 |
|  | (0.004) | (0.004) | (0.004) | (0.004) |
| Woman not working (= 1) | 0.007** | 0.093 | 0.010*** | 0.084 |
|  | (0.003) | (0.124) | (0.003) | (0.129) |
| Generalized residuals |  | -0.016 |  | -0.014 |
|  |  | (0.012) |  | (0.013) |
| Number of observations | 2,061 | 2,061 | 2,061 | 2,061 |

Notes: See notes to Table S4. In columns (2a) and (2b) the instruments are the female employment rate at the municipal level and its variation with respect to 2011.

Table S9 - Estimates without observations from the third round (Waves 1 and 2 only)

A. Dependent variable: 1 if victims of IPV

|  | Any type of IPV | | Psychological IPV | | Sexual and/or physical IPV | |
| --- | --- | --- | --- | --- | --- | --- |
|  | (1a) | (2a) | (1b) | (2b) | (1c) | (2c) |
| Model | Probit | CF | Probit | CF | Probit | CF |
| Average predicted value of dependent variable | 0.0573 | 0.0573 | 0.0535 | 0.0535 | 0.0182 | 0.0182 |
|  | (0.005) | (0.005) | (0.005) | (0.005) | (0.003) | (0.003) |
| *Average Marginal effect* |  |  |  |  |  |  |
| At least one partner lost job (= 1) | 0.039*** | 0.039*** | 0.035*** | 0.035*** | 0.016** | 0.016** |
|  | (0.012) | (0.012) | (0.012) | (0.012) | (0.008) | (0.008) |
| Children (<18 years) in household (= 1) | 0.006 | 0.008 | 0.008 | 0.010 | 0.005 | 0.009 |
|  | (0.011) | (0.011) | (0.011) | (0.011) | (0.006) | (0.007) |
| House with no outside space and non-privacy index >= 1 (= 1) | 0.001 | 0.000 | -0.005 | -0.006 | -0.007 | -0.008 |
|  | (0.017) | (0.017) | (0.016) | (0.016) | (0.008) | (0.008) |
| Woman not working (= 1) | 0.029** | 0.131 | 0.032** | 0.148 | 0.015* | 0.533 |
|  | (0.014) | (0.210) | (0.014) | (0.222) | (0.008) | (0.391) |
| Generalized residuals |  | -0.038 |  | -0.040 |  | -0.062* |
|  |  | (0.062) |  | (0.059) |  | (0.037) |
| Number of observations | 2,028 | 2,028 | 2,028 | 2,028 | 2,028 | 2,028 |

B. Dependent variable: fuzzy indicators of IPV

|  | Any type of IPV | | Psychological IPV | | Sexual and/or physical IPV | |
| --- | --- | --- | --- | --- | --- | --- |
|  | (1a) | (2a) | (1b) | (2b) | (1c) | (2c) |
| Model | Frac. | CF | Frac. | CF | Frac. | CF |
| Average predicted value of dependent variable | 0.00614 | 0.00614 | 0.00919 | 0.00920 | 0.00308 | 0.00309 |
|  | (0.001) | (0.001) | (0.001) | (0.001) | (0.001) | (0.001) |
| *Average marginal effect* |  |  |  |  |  |  |
| At least one partner lost job (= 1) | 0.003 | 0.003 | 0.003 | 0.004 | 0.002 | 0.002 |
|  | (0.002) | (0.002) | (0.003) | (0.003) | (0.001) | (0.002) |
| Children (<18 years) in household (= 1) | 0.004* | 0.005** | 0.005* | 0.007** | 0.002 | 0.003 |
|  | (0.002) | (0.002) | (0.003) | (0.003) | (0.001) | (0.002) |
| House with no outside space and non-privacy index >= 1 (= 1) | -0.000 | -0.001 | 0.001 | -0.000 | -0.002 | -0.002 |
|  | (0.002) | (0.003) | (0.004) | (0.004) | (0.001) | (0.004) |
| Woman not working (= 1) | 0.004 | 0.170 | 0.006* | 0.221 | 0.001 | 0.122 |
|  | (0.002) | (0.203) | (0.003) | (0.238) | (0.002) | (0.206) |
| Generalized residuals |  | -0.016* |  | -0.024* |  | -0.009 |
|  |  | (0.009) |  | (0.013) |  | (0.006) |
| Number of observations | 2,028 | 2,028 | 2,028 | 2,028 | 2,028 | 2,028 |

Notes: See notes to Table S4. Sample restricted to waves 1 and 2 (N excludes wave 3 respondents).

Table S10 - Minimum Detectable Effects (MDEs) compared to Average Marginal Effects (AMEs) (Power = 0.80)

A. Dependent variable: 1 if victims of IPV

| **Outcome** | **Model** | **Variable** | **AME** | **SE** | **MDE (.01)** | **MDE (.05)** | **MDE (.10)** |
| --- | --- | --- | --- | --- | --- | --- | --- |
| Total IPV | Probit | At least one job loss | 0.028 | 0.011 | 0.038 | 0.0310 | **0.027** |
|  |  | Children <18 | 0.022 | 0.010 | 0.034 | 0.0280 | 0.025 |
|  |  | Space deprivation | 0.014 | 0.015 | 0.051 | 0.0420 | 0.037 |
|  |  | Woman Not employed | 0.034 | 0.012 | 0.041 | **0.0340** | **0.030** |
|  | CF | At least one job loss | 0.028 | 0.011 | 0.038 | 0.0310 | **0.027** |
|  |  | Children <18 | 0.025 | 0.010 | 0.034 | 0.0280 | **0.025** |
|  |  | Space deprivation | 0.011 | 0.016 | 0.055 | 0.0450 | 0.040 |
|  |  | Woman Not employed | 0.185 | 0.213 | 0.728 | 0.5960 | 0.529 |
| Psychological | Probit | At least one job loss | 0.025 | 0.010 | 0.034 | 0.0280 | **0.025** |
|  |  | Children <18 | 0.024 | 0.010 | 0.034 | 0.0280 | 0.025 |
|  |  | Space deprivation | 0.009 | 0.014 | 0.048 | 0.0390 | 0.035 |
|  |  | Woman Not employed | 0.036 | 0.012 | 0.041 | **0.0340** | **0.030** |
|  | CF | At least one job loss | 0.025 | 0.010 | 0.034 | 0.0280 | **0.025** |
|  |  | Children <18 | 0.026 | 0.010 | 0.034 | 0.0280 | **0.025** |
|  |  | Space deprivation | 0.006 | 0.015 | 0.051 | 0.0420 | 0.037 |
|  |  | Woman Not employed | 0.191 | 0.220 | 0.752 | 0.6160 | 0.547 |
| Sex./Physical | Probit | At least one job loss | 0.010 | 0.007 | 0.024 | 0.0200 | 0.017 |
|  |  | Children <18 | 0.020 | 0.007 | 0.024 | **0.0200** | **0.017** |
|  |  | Space deprivation | 0.003 | 0.009 | 0.031 | 0.0250 | 0.022 |
|  |  | Woman Not employed | 0.019 | 0.008 | 0.027 | 0.0220 | 0.020 |
|  | CF | At least one job loss | 0.010 | 0.007 | 0.024 | 0.0200 | 0.017 |
|  |  | Children <18 | 0.023 | 0.007 | 0.024 | **0.0200** | **0.017** |
|  |  | Space deprivation | 0.000 | 0.010 | 0.034 | 0.0280 | 0.025 |
|  |  | Woman Not employed | 0.410 | 0.340 | 1.161 | 0.9520 | 0.845 |
| B. Dependent variable: fuzzy indicators of IPV | | | | | |  |  |
| **Outcome** | **Model** | **Variable** | **AME** | **SE** | **MDE (.01)** | **MDE (.05)** | **MDE (.10)** |
| Total IPV | Fracreg | At least one job loss | 0.002 | 0.002 | 0.007 | 0.006 | 0.005 |
|  |  | Children <18 | 0.013 | 0.003 | **0.01** | **0.008** | **0.007** |
|  |  | Space deprivation | 0.006 | 0.004 | 0.014 | 0.011 | 0.01 |
|  |  | Woman Not employed | 0.007 | 0.003 | 0.01 | 0.008 | 0.007 |
|  | CF | At least one job loss | 0.002 | 0.002 | 0.007 | 0.006 | 0.005 |
|  |  | Children <18 | 0.014 | 0.003 | **0.01** | **0.008** | **0.007** |
|  |  | Space deprivation | 0.005 | 0.004 | 0.014 | 0.011 | 0.01 |
|  |  | Woman Not employed | 0.093 | 0.124 | 0.424 | 0.347 | 0.308 |
| Psychological | Fracreg | At least one job loss | 0.002 | 0.003 | 0.01 | 0.008 | 0.007 |
|  |  | Children <18 | 0.016 | 0.004 | **0.014** | **0.011** | **0.01** |
|  |  | Space deprivation | 0.007 | 0.005 | 0.017 | 0.014 | 0.012 |
|  |  | Woman Not employed | 0.011 | 0.004 | 0.014 | 0.011 | **0.01** |
|  | CF | At least one job loss | 0.003 | 0.003 | 0.01 | 0.008 | 0.007 |
|  |  | Children <18 | 0.018 | 0.004 | **0.014** | **0.011** | **0.01** |
|  |  | Space deprivation | 0.006 | 0.005 | 0.017 | 0.014 | 0.012 |
|  |  | Woman Not employed | 0.133 | 0.165 | 0.564 | 0.462 | 0.41 |
| Sex./Physical | Fracreg | At least one job loss | 0.001 | 0.002 | 0.007 | 0.006 | 0.005 |
|  |  | Children <18 | 0.009 | 0.002 | **0.007** | **0.006** | **0.005** |
|  |  | Space deprivation | 0.005 | 0.003 | 0.01 | 0.008 | 0.007 |
|  |  | Woman Not employed | 0.004 | 0.002 | 0.007 | 0.006 | 0.005 |
|  | CF | At least one job loss | 0.002 | 0.002 | 0.007 | 0.006 | 0.005 |
|  |  | Children <18 | 0.01 | 0.003 | 0.01 | **0.008** | **0.007** |
|  |  | Space deprivation | 0.004 | 0.004 | 0.014 | 0.011 | 0.01 |
|  |  | Woman Not employed | 0.051 | 0.088 | 0.301 | 0.246 | 0.219 |

Notes: MDEs are reported for three different significance levels (0.01, 0.05, and 0.10). MDEs smaller than the corresponding AMEs are shown in bold.

*Sensitivity analysis*

The sensitivity tests concern assumptions about errors in the CF model. For our first sensitivity test, we followed [84] who emphasized the importance of comparing CF average partial effects with those derived from joint quasi-maximum likelihood estimation (QMLE). In order to illustrate this test, we need to briefly discuss the difference between CF and JMLE estimations. The JMLE model we estimated was implemented by the Stata user command *cmp* [A1]. The endogenous variable was instrumented as in Equation 4. Indeed, CF and JMLE both use the same reduced form but different assumptions on the conditional distribution of errors (see [83]). For prevalence IPV, the second stage estimates a bivariate probit corresponding to Equation 2. For fuzzy IPV, the second stage estimates the fractional probit regression corresponding to Equation 3. Each pair of equations is estimated simultaneously by a maximum likelihood Seemingly Unrelated Regression (SUR) method for multi-equation models. The latter produces limited-information maximum likelihood estimations which are consistent for recursive systems in which the endogenous variable, as in our case, is on the right-hand side [A1]. Here too, valid standard errors for JMLE estimators were obtained by bootstrapping.

The results reported in this Appendix are reassuring in two important respects (see Table S11). For our covariates of interest (the three stressors), the sign and significance of the JMLE estimates are practically the same as for CF. Moreover, the athanrho statistics, namely the inverse hyperbolic tangent of the correlation coefficient between the error terms of the two equations in the JMLE model, is not significant across IPV specifications. This indicates that not much is lost if the two equations are estimated separately as in the CF model. However, JMLE point estimates turned out to be less precise than CF estimates.

The second test is much more conclusive. Following the procedure in [A2], which Wooldridge himself mentions as an alternative to the CF model, we obtained CF estimates using standard residuals from the first stage regression rather than generalized residuals. The results thus obtained are also shown in Table S12, and they reveal a near overlap with those of the CF model in Table 3.

Table S11 - Estimates comparison with a joint quasi-maximum likelihood estimation, by type of violence and indicator of violence.

| Dependent variable: | 1 if victims of IPV | | | Fuzzy indicators of IPV | | |
| --- | --- | --- | --- | --- | --- | --- |
|  | Any type of IPV | Psychological IPV | Sexual and/or physical IPV | Any type of IPV | Psychological IPV | Sexual and/or physical IPV |
| *Model* | *JM* | *JM* | *JM* | *JM* | *JM* | *JM* |
| *Average Marginal effect* |  |  |  |  |  |  |
| At least one partner lost job (= 1) | 0.033*** | 0.029** | 0.018 | 0.003 | 0.004 | 0.003 |
|  | (0.012) | (0.012) | (0.012) | (0.004) | (0.005) | (0.003) |
| Children (<18 years) in household (= 1) | 0.029** | 0.030** | 0.033*** | 0.019** | 0.024*** | 0.014** |
|  | (0.012) | (0.012) | (0.012) | (0.008) | (0.009) | (0.007) |
| House with no outside space and non-privacy index >= 1 (= 1) | 0.012 | 0.007 | 0.001 | 0.008 | 0.009 | 0.006 |
|  | (0.018) | (0.017) | (0.017) | (0.006) | (0.007) | (0.005) |
| Woman not working (= 1) | 0.278 | 0.228 | 0.222 | 0.046 | 0.065 | 0.027 |
|  | (0.221) | (0.184) | (0.159) | (0.045) | (0.059) | (0.034) |
| atanhrho | -0.693 | -0.582 | -0.851 | -0.445 | -0.463 | -0.448 |
|  | (0.536) | (0.468) | (0.509) | (0.354) | (0.334) | (0.511) |
| Other controls | Yes | Yes | Yes | Yes | Yes | Yes |
| Number of observations | 2,061 | 2,061 | 2,061 | 2,061 | 2,061 | 2,061 |

Notes: See notes to Table S4.

Table S12 - Estimates comparison with Terza, Basu, and Rathouz CF’s approach and a joint quasi-maximum likelihood estimation

A. Dependent variable: 1 if victims of IPV

|  | Any type of IPV | | |
| --- | --- | --- | --- |
|  | (1a) | (2a) | (3a) |
| *Model* | *CF1* | *CF2* | *JM* |
| Average predicted value of dependent | 0.057 | 0.057 | 0.099 |
|  | (0.005) | (0.005) | (0.037) |
| *Average Marginal effect* |  |  |  |
| At least one partner lost job (= 1) | 0.028*** | 0.028*** | 0.033*** |
|  | (0.011) | (0.011) | (0.012) |
| Children (<18 years) in household (= 1) | 0.025** | 0.024** | 0.029** |
|  | (0.010) | (0.011) | (0.012) |
| House with no outside space and non-privacy index >= 1 (= 1) | 0.011 | 0.012 | 0.012 |
|  | (0.016) | (0.016) | (0.018) |
| Woman not working (= 1) | 0.185 | 0.144 | 0.278 |
|  | (0.213) | (0.241) | (0.221) |
| First stage generalised residuals | -0.051 |  |  |
|  | (0.054) |  |  |
| First stage residuals |  | -0.069 |  |
|  |  | (0.119) |  |
| atanhrho |  |  | -0.693 |
|  |  |  | (0.536) |

B. Dependent variable: fuzzy indicators of IPV

|  | Any type of IPV | | |
| --- | --- | --- | --- |
|  | (1a) | (2a) | (3a) |
| *Model* | *CF1* | *CF2* | *JM* |
| Average predicted value of dependent | 0.011 | 0.011 | 0.019 |
|  | (0.001) | (0.001) | (0.009) |
|  |  |  |  |
| *Average Marginal effect* |  |  |  |
| At least one partner lost job (= 1) | 0.002 | 0.002 | 0.003 |
|  | (0.002) | (0.002) | (0.004) |
| Children (<18 years) in household (= 1) | 0.014*** | 0.014*** | 0.019** |
|  | (0.003) | (0.003) | (0.008) |
| House with no outside space and non-privacy index >= 1 (= 1) | 0.005 | 0.005 | 0.008 |
|  | (0.004) | (0.004) | (0.006) |
| Woman not working (= 1) | 0.093 | 0.162 | 0.046 |
|  | (0.124) | (0.207) | (0.045) |
| First stage generalized residuals | -0.016 |  |  |
|  | (0.012) |  |  |
| First stage residuals |  | -0.037 |  |
|  |  | (0.024) |  |
| atanhrho^1^ |  |  | -0.445 |
|  |  |  | (0.354) |
| Number of observations | 2,061 | 2,061 | 2,061 |

Notes: See notes to Table S4. CF1 stands for Control Function as proposed by [83]. CF2 for Control Function as proposed by [A1], and JM to joint models (joint QMLE). 1. The variable atanhrho refers to arc-hyperbolic tangents of the ρ (see [A1]).

# Appendix References

**A****1.** Roodman D. Fitting Fully Observed Recursive Mixed-process Models with CMP. Stata J. 2011

**A****2.** Terza JV, Basu A, Rathouz PJ. Two-stage residual inclusion estimation: addressing endogeneity in health econometric modeling. J Health Econ. 2008;27(3):531-543.
