# Supplementary material for: Intimate partner violence during lockdown in Tuscany, Italy: Economic or confinement-related shocks?
Source: PLoS One. 2026 Jun 24;21(6):e0349889. doi: 10.1371/journal.pone.0349889 (PMC13293386; doi:10.1371/journal.pone.0349889)
Supplement: Attachment — Submitted filename: Response to Reviewers.docx [file pone.0349889.s005.docx]

*Dear Reviewers*

*Thank you for the insightful comments that have greatly helped us improve the paper. Please find below our answer in italics under each comment.*

**Reviewer #1**: Thank you so much for the opportunity to review the manuscript titled “Intimate partner violence during lockdown in Tuscany, Italy: economic shock or confinement-related stressors?” The manuscript is well structured and innovative presenting an under-explored area of IPV during the pandemic. The study uses a representative dataset and introduction of a IPV index adds significant value to the field. However, minor revisions and clarifications are needed particularly regarding rigor and statistical transparency.

*We are delighted that you appreciate the novelties our paper brings. We were able to address all your requests, except one.*

1. The key result that parental overburden had the highest impact on IPV risk is important. However, the absolute number of IPV cases is relatively low (approximately 150 out of 2,061), which raises concerns about statistical power. This is especially critical in models estimating effects for subtypes, for example, sexual violence with approximately 34 cases. The authors should provide a power analysis or at least discuss the limitations of inference due to small sample size.

*Section 6.3 now includes a paragraph comparing the average marginal effects (AMEs) of parental overburden, job loss, and confinement in crowded space with the corresponding Minimum Detectable Effects (MDEs). The actual values are reported in Appendix S3 (Table S10) while the paragraph acknowledges that we cannot rule out underpower for two of our effects of interest, albeit within the limitations of ex-post power analysis.*

2. It is unclear whether interaction terms were tested, which could reveal important moderation effects consistent with stress and gender norm theories. This should be explored or discussed.

*We particularly appreciated this suggestion! Section 6 now includes subsection 6.2 to explore the role gender norms may have played in moderating the impact of parental overburden. Given that the PANGEA survey did not investigate attitudes, we proxied adherence to traditional gender norms with the woman’s foreign status and with her partner’s low education. We used these proxies to construct two interaction terms with parental overburden, which we added to our set of covariates. The findings seem to support the idea that adherence to traditional gender norms exacerbated the intensity and severity of IPV rather than affecting its prevalence. However, we recognize that the use of proxies warrants caution in interpreting the results of the interactions.*

3. The process for adjusting sampling weights due to oversampling of abused women in round 3 is described, but the assumption that abuse prevalence remains unchanged may bias results. Consider conducting sensitivity analyses (if feasible), excluding round 3 data or using propensity score adjustments to compare subsamples.

*We performed a robustness check by excluding all third-round observations and included an additional paragraph to illustrate and discuss the results. Although dropping these observations alters the pattern of significance of our main estimates, it does not, in our view, question the hierarchy of effects we found using the complete sample.*

4. Acknowledge potential recall biases, especially in telephone surveys, as well as differences in disclosure by IPV type (e.g., psychological abuse underreporting vs. physical abuse).

*In section 4.2 we now explicitly mention recall bias in connection with the possibility that the psychological abuse was underreported by participants in the PANGEA survey.*

5. The terms “shock,” “stressor,” and “trigger” are used somewhat interchangeably. Consider defining these terms clearly and using them consistently to enhance clarity.

*Again, many thanks for this suggestion! We opted in favour of using shocks as consistently as possible, given that the term is closely associated with the occurrence of unexpected events.*

6. Time of data collection (2021–2023) spanned different lockdown phases; subgroup analysis by lockdown phase may reveal temporal variations (if feasible)

*Unfortunately, this was not feasible.*

7. Please perform a through editorial check as there were typographical errors (for example, use of period in between the sentence)

*Done! Hopefully the text reads much better now. Please note that we moved the two paragraphs discussing sensitivity tests from the main text to the Appendix in order to make room for the revisions (Tables S11 and S12 and related discussion in Appendix 3).*

**Reviewer #2**: The document is evidence of the increasing difficulty in accessing representative information on social and economic phenomena, which paradoxically is experienced in increasingly connected communities. In fact, most of the work done by the authors serves to control the limited sensitivity of the available data. I find the use of fuzzy variables in statistical analysis particularly interesting, among other reasons, because it seems to me the most coherent result and opens up new analytical possibilities. I must point out that the reference to previous works is made using the citation number, and I believe that this is not the standard for PLOS ONE. I suggest reviewing the editorial guidelines.

*We gratefully acknowledge your remark that the use of fuzzy variables opens new analytical possibilities in the study of violence against women. This is indeed something to which we hope the paper will contribute.*

*Thank you also for advising us to check the editorial guidelines on references, which we did. In our understanding, the style we used is what PLOS recommends. The journal uses the numbered citation (citation-sequence) method and recommends that authors cite the reference number in square brackets in the text (e.g., “We used the techniques developed by our colleagues [19] to analyze the data”).*
