# Supplementary material for: Intimate partner violence during lockdown in Tuscany, Italy: Economic or confinement-related shocks?
Source: PLoS One. 2026 Jun 24;21(6):e0349889. doi: 10.1371/journal.pone.0349889 (PMC13293386; doi:10.1371/journal.pone.0349889)
Supplement: Peer review history

# Peer review: Decision Letter 0

**DOI:** 10.1371/journal.pone.0349889.r001

**Authors:** Adetayo Olorunlana

25 Feb 2026

PONE-D-25-33985

Intimate partner violence during lockdown in Tuscany, Italy: economic shock or confinement-related stressors?

PLOS One

Dear Dr. Flores Tavares,

Thank you for submitting your manuscript to PLOS ONE. After careful consideration, we feel that it has merit but does not fully meet PLOS ONE’s publication criteria as it currently stands. Therefore, we invite you to submit a revised version of the manuscript that addresses the points raised during the review process.

Please submit your revised manuscript by Apr 11 2026 11:59PM. If you will need more time than this to complete your revisions, please reply to this message or contact the journal office at plosone@plos.org. When you're ready to submit your revision, log on to https://www.editorialmanager.com/pone/ and select the 'Submissions Needing Revision' folder to locate your manuscript file.

Please include the following items when submitting your revised manuscript:

- A letter that responds to each point raised by the academic editor and reviewer(s). You should upload this letter as a separate file labeled 'Response to Reviewers'.
- A marked-up copy of your manuscript that highlights changes made to the original version. You should upload this as a separate file labeled 'Revised Manuscript with Track Changes'.
- An unmarked version of your revised paper without tracked changes. You should upload this as a separate file labeled 'Manuscript'.

-->If you would like to make changes to your financial disclosure, please include your updated statement in your cover letter. Guidelines for resubmitting your figure files are available below the reviewer comments at the end of this letter.

If applicable, we recommend that you deposit your laboratory protocols in protocols.io to enhance the reproducibility of your results. Protocols.io assigns your protocol its own identifier (DOI) so that it can be cited independently in the future. For instructions see: https://journals.plos.org/plosone/s/submission-guidelines#loc-laboratory-protocols. Additionally, PLOS ONE offers an option for publishing peer-reviewed Lab Protocol articles, which describe protocols hosted on protocols.io. Read more information on sharing protocols at https://plos.org/protocols?utm_medium=editorial-email&utm_source=authorletters&utm_campaign=protocols.

We look forward to receiving your revised manuscript.

Kind regards,

Adetayo Olorunlana, Ph.D.

Academic Editor

PLOS One

**Journal Requirements:**

When submitting your revision, we need you to address these additional requirements.

1. Please ensure that your manuscript meets PLOS ONE's style requirements, including those for file naming. The PLOS ONE style templates can be found at

https://journals.plos.org/plosone/s/file?id=wjVg/PLOSOne_formatting_sample_main_body.pdf and

https://journals.plos.org/plosone/s/file?id=ba62/PLOSOne_formatting_sample_title_authors_affiliations.pdf

2. Thank you for stating the following financial disclosure:

“This research is part of the PANGEA project (“PANdemic GEnder violence participatory Assessment”), funded by Regione Toscana.”

Please state what role the funders took in the study.  If the funders had no role, please state: "The funders had no role in study design, data collection and analysis, decision to publish, or preparation of the manuscript."

If this statement is not correct you must amend it as needed.

Please include this amended Role of Funder statement in your cover letter; we will change the online submission form on your behalf.

3. In the online submission form you indicate that your data is not available for proprietary reasons and have provided a contact point for accessing this data. Please note that your current contact point is a co-author on this manuscript. According to our Data Policy, the contact point must not be an author on the manuscript and must be an institutional contact, ideally not an individual. Please revise your data statement to a non-author institutional point of contact, such as a data access or ethics committee, and send this to us via return email. Please also include contact information for the third party organization, and please include the full citation of where the data can be found.

4. If the reviewer comments include a recommendation to cite specific previously published works, please review and evaluate these publications to determine whether they are relevant and should be cited. There is no requirement to cite these works unless the editor has indicated otherwise.

5. Please review your reference list to ensure that it is complete and correct. If you have cited papers that have been retracted, please include the rationale for doing so in the manuscript text, or remove these references and replace them with relevant current references. Any changes to the reference list should be mentioned in the rebuttal letter that accompanies your revised manuscript. If you need to cite a retracted article, indicate the article’s retracted status in the References list and also include a citation and full reference for the retraction notice.

[Note: HTML markup is below. Please do not edit.]

Reviewers' comments:

Reviewer's Responses to Questions

**Comments to the Author**

1. Is the manuscript technically sound, and do the data support the conclusions?

The manuscript must describe a technically sound piece of scientific research with data that supports the conclusions. Experiments must have been conducted rigorously, with appropriate controls, replication, and sample sizes. The conclusions must be drawn appropriately based on the data presented.

Reviewer #1: Yes

Reviewer #2: Yes

**********

2. Has the statistical analysis been performed appropriately and rigorously?

Reviewer #1: Yes

Reviewer #2: Yes

**********

3. Have the authors made all data underlying the findings in their manuscript fully available?

The PLOS Data policy requires authors to make all data underlying the findings described in their manuscript fully available without restriction, with rare exception (please refer to the Data Availability Statement in the manuscript PDF file). The data should be provided as part of the manuscript or its supporting information, or deposited to a public repository. For example, in addition to summary statistics, the data points behind means, medians and variance measures should be available. If there are restrictions on publicly sharing data—e.g. participant privacy or use of data from a third party—those must be specified.

Reviewer #1: Yes

Reviewer #2: No

**********

4. Is the manuscript presented in an intelligible fashion and written in standard English?

PLOS ONE does not copyedit accepted manuscripts, so the language in submitted articles must be clear, correct, and unambiguous. Any typographical or grammatical errors should be corrected at revision, so please note any specific errors here.

Reviewer #1: Yes

Reviewer #2: No

**********

5. Review Comments to the Author

Please use the space provided to explain your answers to the questions above. You may also include additional comments for the author, including concerns about dual publication, research ethics, or publication ethics. (Please upload your review as an attachment if it exceeds 20,000 characters)

Reviewer #1: Thank you so much for the opportunity to review the manuscript titled “Intimate partner violence during lockdown in Tuscany, Italy: economic shock or confinement-related stressors?” The manuscript is well structured and innovative presenting an under-explored area of IPV during the pandemic. The study uses a representative dataset and introduction of a IPV index adds significant value to the field. However, minor revisions and clarifications are needed particularly regarding rigor and statistical transparency.

1. The key result that parental overburden had the highest impact on IPV risk is important. However, the absolute number of IPV cases is relatively low (approximately 150 out of 2,061), which raises concerns about statistical power. This is especially critical in models estimating effects for subtypes, for example, sexual violence with approximately 34 cases. The authors should provide a power analysis or at least discuss the limitations of inference due to small sample size.

2. It is unclear whether interaction terms were tested, which could reveal important moderation effects consistent with stress and gender norm theories. This should be explored or discussed.

3. The process for adjusting sampling weights due to oversampling of abused women in round 3 is described, but the assumption that abuse prevalence remains unchanged may bias results. Consider conducting sensitivity analyses (if feasible), excluding round 3 data or using propensity score adjustments to compare subsamples.

4. Acknowledge potential recall biases, especially in telephone surveys, as well as differences in disclosure by IPV type (e.g., psychological abuse underreporting vs. physical abuse).

5. The terms “shock,” “stressor,” and “trigger” are used somewhat interchangeably. Consider defining these terms clearly and using them consistently to enhance clarity.

6. Time of data collection (2021–2023) spanned different lockdown phases; subgroup analysis by lockdown phase may reveal temporal variations (if feasible)

7. Please perform a through editorial check as there were typographical errors (for example, use of period in between the sentence)

Reviewer #2: The document is evidence of the increasing difficulty in accessing representative information on social and economic phenomena, which paradoxically is experienced in increasingly connected communities. In fact, most of the work done by the authors serves to control the limited sensitivity of the available data. I find the use of fuzzy variables in statistical analysis particularly interesting, among other reasons, because it seems to me the most coherent result and opens up new analytical possibilities. I must point out that the reference to previous works is made using the citation number, and I believe that this is not the standard for PLOS ONE. I suggest reviewing the editorial guidelines.

**********

6. PLOS authors have the option to publish the peer review history of their article (what does this mean?). If published, this will include your full peer review and any attached files.

If you choose “no”, your identity will remain anonymous but your review may still be made public.

**Do you want your identity to be public for this peer review?**  For information about this choice, including consent withdrawal, please see our Privacy Policy.

Reviewer #1: No

Reviewer #2: No

**********

[NOTE: If reviewer comments were submitted as an attachment file, they will be attached to this email and accessible via the submission site. Please log into your account, locate the manuscript record, and check for the action link "View Attachments". If this link does not appear, there are no attachment files.]

To ensure your figures meet our technical requirements, please review our figure guidelines: https://journals.plos.org/plosone/s/figures

You may also use PLOS’s free figure tool, NAAS, to help you prepare publication quality figures: https://journals.plos.org/plosone/s/figures#loc-tools-for-figure-preparation.

NAAS will assess whether your figures meet our technical requirements by comparing each figure against our figure specifications.

# Author comment: Author response to Decision Letter 1

**DOI:** 10.1371/journal.pone.0349889.r002

10 Apr 2026

Dear Reviewers

Thank you for the insightful comments that have greatly helped us improve the paper. Please find below our answer under each comment.

Reviewer #1: Thank you so much for the opportunity to review the manuscript titled “Intimate partner violence during lockdown in Tuscany, Italy: economic shock or confinement-related stressors?” The manuscript is well structured and innovative presenting an under-explored area of IPV during the pandemic. The study uses a representative dataset and introduction of a IPV index adds significant value to the field. However, minor revisions and clarifications are needed particularly regarding rigor and statistical transparency.

Response: We are delighted that you appreciate the novelties our paper brings. We were able to address all your requests, except one.

1. The key result that parental overburden had the highest impact on IPV risk is important. However, the absolute number of IPV cases is relatively low (approximately 150 out of 2,061), which raises concerns about statistical power. This is especially critical in models estimating effects for subtypes, for example, sexual violence with approximately 34 cases. The authors should provide a power analysis or at least discuss the limitations of inference due to small sample size.

Response: Section 6.3 now includes a paragraph comparing the average marginal effects (AMEs) of parental overburden, job loss, and confinement in crowded space with the corresponding Minimum Detectable Effects (MDEs). The actual values are reported in Appendix S3 (Table S10) while the paragraph acknowledges that we cannot rule out underpower for two of our effects of interest, albeit within the limitations of ex-post power analysis.

2. It is unclear whether interaction terms were tested, which could reveal important moderation effects consistent with stress and gender norm theories. This should be explored or discussed.

Response: We particularly appreciated this suggestion! Section 6 now includes subsection 6.2 to explore the role gender norms may have played in moderating the impact of parental overburden. Given that the PANGEA survey did not investigate attitudes, we proxied adherence to traditional gender norms with the woman’s foreign status and with her partner’s low education. We used these proxies to construct two interaction terms with parental overburden, which we added to our set of covariates. The findings seem to support the idea that adherence to traditional gender norms exacerbated the intensity and severity of IPV rather than affecting its prevalence. However, we recognize that the use of proxies warrants caution in interpreting the results of the interactions.

3. The process for adjusting sampling weights due to oversampling of abused women in round 3 is described, but the assumption that abuse prevalence remains unchanged may bias results. Consider conducting sensitivity analyses (if feasible), excluding round 3 data or using propensity score adjustments to compare subsamples.

Response: We performed a robustness check by excluding all third-round observations and included an additional paragraph to illustrate and discuss the results. Although dropping these observations alters the pattern of significance of our main estimates, it does not, in our view, question the hierarchy of effects we found using the complete sample.

4. Acknowledge potential recall biases, especially in telephone surveys, as well as differences in disclosure by IPV type (e.g., psychological abuse underreporting vs. physical abuse).

Response: In section 4.2 we now explicitly mention recall bias in connection with the possibility that the psychological abuse was underreported by participants in the PANGEA survey.

5. The terms “shock,” “stressor,” and “trigger” are used somewhat interchangeably. Consider defining these terms clearly and using them consistently to enhance clarity.

Response: Again, many thanks for this suggestion! We opted in favour of using shocks as consistently as possible, given that the term is closely associated with the occurrence of unexpected events.

6. Time of data collection (2021–2023) spanned different lockdown phases; subgroup analysis by lockdown phase may reveal temporal variations (if feasible)

Response: Unfortunately, this was not feasible.

7. Please perform a through editorial check as there were typographical errors (for example, use of period in between the sentence)

Response: Done! Hopefully, the text reads much better now. Please note that we moved the two paragraphs discussing sensitivity tests from the main text to the Appendix in order to make room for the revisions (Tables S11 and S12 and related discussion in Appendix 3).

Reviewer #2: The document is evidence of the increasing difficulty in accessing representative information on social and economic phenomena, which paradoxically is experienced in increasingly connected communities. In fact, most of the work done by the authors serves to control the limited sensitivity of the available data. I find the use of fuzzy variables in statistical analysis particularly interesting, among other reasons, because it seems to me the most coherent result and opens up new analytical possibilities. I must point out that the reference to previous works is made using the citation number, and I believe that this is not the standard for PLOS ONE. I suggest reviewing the editorial guidelines.

We gratefully acknowledge your remark that the use of fuzzy variables opens new analytical possibilities in the study of violence against women. This is indeed something to which we hope the paper will contribute.

Response: Thank you also for advising us to check the editorial guidelines on references, which we did. In our understanding, the style we used is what PLOS recommends. The journal uses the numbered citation (citation-sequence) method and recommends that authors cite the reference number in square brackets in the text (e.g., “We used the techniques developed by our colleagues [19] to analyze the data”).

Attachment Submitted filename: Response to Reviewers.docx

# Peer review: Decision Letter 1

**DOI:** 10.1371/journal.pone.0349889.r003

**Authors:** Adetayo Olorunlana

6 May 2026

Intimate partner violence during lockdown in Tuscany, Italy: economic or confinement-related shocks?

PONE-D-25-33985R1

Dear Dr. Flores Tavares,

We’re pleased to inform you that your manuscript has been judged scientifically suitable for publication and will be formally accepted for publication once it meets all outstanding technical requirements.

Within one week, you’ll receive an e-mail detailing the required amendments. When these have been addressed, you’ll receive a formal acceptance letter and your manuscript will be scheduled for publication.

An invoice will be generated when your article is formally accepted. Please note, if your institution has a publishing partnership with PLOS and your article meets the relevant criteria, all or part of your publication costs will be covered. Please make sure your user information is up-to-date by logging into Editorial Manager at Editorial Manager® and clicking the ‘Update My Information' link at the top of the page. For questions related to billing, please contact billing support.

If your institution or institutions have a press office, please notify them about your upcoming paper to help maximize its impact. If they’ll be preparing press materials, please inform our press team as soon as possible -- no later than 48 hours after receiving the formal acceptance. Your manuscript will remain under strict press embargo until 2 pm Eastern Time on the date of publication. For more information, please contact onepress@plos.org.

Kind regards,

Adetayo Olorunlana, Ph.D.

Academic Editor

PLOS One

Additional Editor Comments (optional):

Reviewers' comments:

Reviewer's Responses to Questions

**Comments to the Author**

1. If the authors have adequately addressed your comments raised in a previous round of review and you feel that this manuscript is now acceptable for publication, you may indicate that here to bypass the “Comments to the Author” section, enter your conflict of interest statement in the “Confidential to Editor” section, and submit your "Accept" recommendation.

Reviewer #2: All comments have been addressed

**********

2. Is the manuscript technically sound, and do the data support the conclusions?

The manuscript must describe a technically sound piece of scientific research with data that supports the conclusions. Experiments must have been conducted rigorously, with appropriate controls, replication, and sample sizes. The conclusions must be drawn appropriately based on the data presented.

Reviewer #2: Yes

**********

3. Has the statistical analysis been performed appropriately and rigorously?

Reviewer #2: Yes

**********

4. Have the authors made all data underlying the findings in their manuscript fully available?

The PLOS Data policy requires authors to make all data underlying the findings described in their manuscript fully available without restriction, with rare exception (please refer to the Data Availability Statement in the manuscript PDF file). The data should be provided as part of the manuscript or its supporting information, or deposited to a public repository. For example, in addition to summary statistics, the data points behind means, medians and variance measures should be available. If there are restrictions on publicly sharing data—e.g. participant privacy or use of data from a third party—those must be specified.

Reviewer #2: Yes

**********

5. Is the manuscript presented in an intelligible fashion and written in standard English?

PLOS ONE does not copyedit accepted manuscripts, so the language in submitted articles must be clear, correct, and unambiguous. Any typographical or grammatical errors should be corrected at revision, so please note any specific errors here.

Reviewer #2: Yes

**********

6. Review Comments to the Author

Please use the space provided to explain your answers to the questions above. You may also include additional comments for the author, including concerns about dual publication, research ethics, or publication ethics. (Please upload your review as an attachment if it exceeds 20,000 characters)

Reviewer #2: The authors carried out a detailed and well-documented review. They managed to address all the observations made by the anonymous reviewers and provided clear and coherent explanations to the questions raised. The honesty of the authors should be recognised and valued, as they pointed out the limitations of the data and the scope of the conclusions reached.

**********

7. PLOS authors have the option to publish the peer review history of their article (what does this mean?). If published, this will include your full peer review and any attached files.

If you choose “no”, your identity will remain anonymous but your review may still be made public.

**Do you want your identity to be public for this peer review?**  For information about this choice, including consent withdrawal, please see our Privacy Policy.

Reviewer #2: No

**********

# Editor report: Acceptance letter

**DOI:** 10.1371/journal.pone.0349889.r004

**Authors:** Adetayo Olorunlana

PONE-D-25-33985R1

PLOS One

Dear Dr. Flores Tavares,

I'm pleased to inform you that your manuscript has been deemed suitable for publication in PLOS One. Congratulations! Your manuscript is now being handed over to our production team.

At this stage, our production department will prepare your paper for publication. This includes ensuring the following:

* All references, tables, and figures are properly cited

* All relevant supporting information is included in the manuscript submission,

* There are no issues that prevent the paper from being properly typeset

You will receive further instructions from the production team, including instructions on how to review your proof when it is ready. Please keep in mind that we are working through a large volume of accepted articles, so please give us a few days to review your paper and let you know the next and final steps.

Lastly, if your institution or institutions have a press office, please let them know about your upcoming paper now to help maximize its impact. If they'll be preparing press materials, please inform our press team within the next 48 hours. Your manuscript will remain under strict press embargo until 2 pm Eastern Time on the date of publication. For more information, please contact onepress@plos.org.

You will receive an invoice from PLOS for your publication fee after your manuscript has reached the completed accept phase. If you receive an email requesting payment before acceptance or for any other service, this may be a phishing scheme. Learn how to identify phishing emails and protect your accounts at https://explore.plos.org/phishing.

If we can help with anything else, please email us at customercare@plos.org.

Thank you for submitting your work to PLOS ONE and supporting open access.

Kind regards,

PLOS ONE Editorial Office Staff

on behalf of

Professor Adetayo Olorunlana

Academic Editor

PLOS One
